# Analysis of Rare, Exonic Variation amongst Subjects with Autism Spectrum Disorders and Population Controls

**DOI:** 10.1371/journal.pgen.1003443

**Published:** 2013-04-11

**Authors:** Li Liu, Aniko Sabo, Benjamin M. Neale, Uma Nagaswamy, Christine Stevens, Elaine Lim, Corneliu A. Bodea, Donna Muzny, Jeffrey G. Reid, Eric Banks, Hillary Coon, Mark DePristo, Huyen Dinh, Tim Fennel, Jason Flannick, Stacey Gabriel, Kiran Garimella, Shannon Gross, Alicia Hawes, Lora Lewis, Vladimir Makarov, Jared Maguire, Irene Newsham, Ryan Poplin, Stephan Ripke, Khalid Shakir, Kaitlin E. Samocha, Yuanqing Wu, Eric Boerwinkle, Joseph D. Buxbaum, Edwin H. Cook, Bernie Devlin, Gerard D. Schellenberg, James S. Sutcliffe, Mark J. Daly, Richard A. Gibbs, Kathryn Roeder

**Affiliations:** 1 Department of Statistics, Carnegie Mellon University, Pittsburgh, Pennsylvania, United States of America; 2 Human Genome Sequencing Center, Baylor College of Medicine, Houston, Texas, United States of America; 3 Analytic and Translational Genetics Unit, Department of Medicine, Massachusetts General Hospital and Harvard Medical School, Boston, Massachusetts, United States of America; 4 Program in Medical and Population Genetics, Broad Institute of Harvard and MIT, Cambridge, Massachusetts, United States of America; 5 Department of Psychiatry, University of Utah, Salt Lake City, Utah, United States of America; 6 Seaver Autism Center for Research and Treatment, Mount Sinai School of Medicine, New York, New York, United States of America; 7 Department of Psychiatry, Mount Sinai School of Medicine, New York, New York, United States of America; 8 University of Texas MD Anderson Cancer Center, Houston, Texas, United States of America; 9 Human Genetics Center, University of Texas Health Science Center at Houston, Houston, Texas, United States of America; 10 Department of Genetics and Genomic Sciences, Mount Sinai School of Medicine, New York, New York, United States of America; 11 Friedman Brain Institute, Mount Sinai School of Medicine, New York, New York United States of America; 12 Department of Psychiatry, University of Illinois at Chicago, Chicago, Illinois, United States of America; 13 Department of Psychiatry, University of Pittsburgh School of Medicine, Pittsburgh, Pennsylvania, United States of America; 14 Pathology and Laboratory Medicine, Perelman School of Medicine, University of Pennsylvania, Philadelphia, Pennsylvania, United States of America; 15 Vanderbilt Brain Institute, Department of Molecular Physiology and Biophysics and Department of Psychiatry, Vanderbilt University, Nashville, Tennessee, United States of America; 16 Ray and Stephanie Lane Center for Computational Biology, Carnegie Mellon University, Pittsburgh, Pennsylvania, United States of America; Wellcome Trust Sanger Institute, United Kingdom

## Abstract

We report on results from whole-exome sequencing (WES) of 1,039 subjects diagnosed with autism spectrum disorders (ASD) and 870 controls selected from the NIMH repository to be of similar ancestry to cases. The WES data came from two centers using different methods to produce sequence and to call variants from it. Therefore, an initial goal was to ensure the distribution of rare variation was similar for data from different centers. This proved straightforward by filtering called variants by fraction of missing data, read depth, and balance of alternative to reference reads. Results were evaluated using seven samples sequenced at both centers and by results from the association study. Next we addressed how the data and/or results from the centers should be combined. Gene-based analyses of association was an obvious choice, but should statistics for association be combined across centers (meta-analysis) or should data be combined and then analyzed (mega-analysis)? Because of the nature of many gene-based tests, we showed by theory and simulations that mega-analysis has better power than meta-analysis. Finally, before analyzing the data for association, we explored the impact of population structure on rare variant analysis in these data. Like other recent studies, we found evidence that population structure can confound case-control studies by the clustering of rare variants in ancestry space; yet, unlike some recent studies, for these data we found that principal component-based analyses were sufficient to control for ancestry and produce test statistics with appropriate distributions. After using a variety of gene-based tests and both meta- and mega-analysis, we found no new risk genes for ASD in this sample. Our results suggest that standard gene-based tests will require much larger samples of cases and controls before being effective for gene discovery, even for a disorder like ASD.

## Author Summary

This study evaluates association of rare variants and autism spectrum disorders (ASD) in case and control samples sequenced by two centers. Before doing association analyses, we studied how to combine information across studies. We first harmonized the whole-exome sequence (WES) data, across centers, in terms of the distribution of rare variation. Key features included filtering called variants by fraction of missing data, read depth, and balance of alternative to reference reads. After filtering, the vast majority of variants calls from seven samples sequenced at both centers matched. We also evaluated whether one should combine summary statistics from data from each center (meta-analysis) or combine data and analyze it together (mega-analysis). For many gene-based tests, we showed that mega-analysis yields more power. After quality control of data from 1,039 ASD cases and 870 controls and a range of analyses, no gene showed exome-wide evidence of significant association. Our results comport with recent results demonstrating that hundreds of genes affect risk for ASD; they suggest that rare risk variants are scattered across these many genes, and thus larger samples will be required to identify those genes.

## Introduction

Common and rare variants are important constituents of the genetic architecture of Autism Spectrum Disorders (ASD) [1]–[12]. Nonetheless analysis of rare variants has produced the vast majority of findings that implicate certain genes as playing a role in liability for ASD (i.e., ASD genes). Because of the promise of identifying novel ASD genes via rare variants, and the potential downstream implications regarding treatment, an ambitious exome sequencing study has been implemented including nearly 2000 case and control subjects sequenced at two genomic centers. Exome sequencing studies of complex traits have shown success in candidate gene studies [13]–[18]; however, most published candidate gene studies have not reported a p-value small enough to attain exome-wide significance [19].

For rare variants, even if effects are strong, single variant tests typically have little power. Rare variants have to be combined in some way, such as within a gene or across genes, for an association test to reach sufficient power. Hence statistical tests examine the cumulative effects over the observed rare variants in the target set. A number of statistical methods to test for association with rare variants are now available. Several of these tests fall into the category of burden tests in that they assess association with a “super-variant” [20]–[24]. Each of these burden methods assumes variants impact the phenotype in a common direction. Rather than aggregating variants, another class of methods, including C-alpha [25] and SKAT [26], look for an unusual distribution of rare variation among cases and controls.

Power of the test is determined by the number of causal variants in the gene, the size of the corresponding effects, and the sample size. Assuming that the rarest variants are likely to have the largest effects, it is challenging to amass substantial evidence for association without a large sample size. Based on extrapolation of effect sizes and frequencies from published studies [19], the results indicate that thousands of individuals are required to obtain genome wide significance.

In this ARRA autism sequencing consortium (AASC) study, data have been produced by two sequencing centers (Baylor College of Medicine and Broad Institute) and by different exome capture methods, different sequencing platforms and different pre-processing alignment and variant calling methods. Therefore the coverage and quality of these data sets varies. Nonetheless, as we show in the sequel, these data can be harmonized using standard filtering criteria. Given the distinct data sources, the most effective way of testing for association is unclear. Following in the tradition of association studies, meta-analysis is a natural option [27]. With this approach we can perform the analysis on each data set separately and then combine p-values using the weighted Z-score method. Alternatively, after filtering to homogenize data, we can combine the two data sets directly and perform mega-analysis. Meta-analysis has the advantage of permitting and adjusting for heterogeneity between samples [28]. All other things being equal, this is the preferred choice. On the other hand, if the power of mega-analysis is better, then this option is worth pursuing. In this report we show that mega-analysis is the more powerful procedure for gene-based tests, such as SKAT [26], a result that might be counter-intuitive given the well-known efficiency of meta-analysis for tests of linear form such as logistic regression. For these data we also find that population structure appears to be corrected for by using principal components analysis [29].

After quality control and controlling for ancestry, analysis of AASC data reveals no clear-cut associations, including associations in *known* ASD genes. We conclude that rare variants affecting risk are not clustering in a small number of genes, supporting recent results from *de novo* single nucleotide and copy number studies showing that hundreds of genes in the genome affect risk for ASD [4]–[6], [8].

## Results

### Harmonizing Calls of Genotype across Sequencing Platforms

The AASC whole-exome sequencing data included 1039 ASD subjects of European ancestry and 870 controls of similar ancestry. Approximately half of the samples were sequenced using the Solid platform and called with AtlasSNP 2 [30] (Baylor: 505 cases, 491 controls) and the remainder were sequenced using the Illumina platform and called with GATK [31] (Broad: 534 cases, 379 controls).

We considered 6 filters to make these data sets more similar in terms of the distribution of variants in the exome. Filters were sequential in their stringency for including a variant: Filter PASS included variants that pass the baseline filter of GATK; Filter MISS excluded any variant with more than 10% missingness; Four additional filters placed increasingly stringent requirements on depth and balance of reference and alternative allele calls (see Methods). If not otherwise stated, results for analyses were based on the least stringent of these: Filter DpBal, which filters by missingness 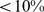, depth 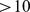, balance 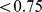 for Broad and 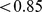 for Baylor.

Seven control samples were sequenced by both centers, facilitating an independent comparison of cross platform calls and an evaluation of the filtering process. To do so, we identified all rare (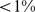), non-synonymous variants located in at least one of the two data sets. Using Filter PASS, in total, these seven samples had 337,478 calls and only .039% of them were mismatched. With Filter DpBal, 290,426 calls remained and .017% of them were mismatched (Table S1). Of the heterozygotes called by one center, but not the other, the mismatch rate was not symmetric: 9 heterozygotes were called by Baylor, but not by Broad, while 42 heterozygotes were called by Broad, but not by Baylor. On closer inspection, many of these heterozygotes did appear to be present; however, one of the variant callers was not confident enough to make the call. Application of the stricter filters (B–D) led to the removal of many of the heterozygous calls for which the callers matched without further improvement in the mismatch rate. For instance, with Filter D only 65% of the matching heterozygous calls from Filter PASS were preserved compared to 85% for Filter DpBal.

**Figure 1:**
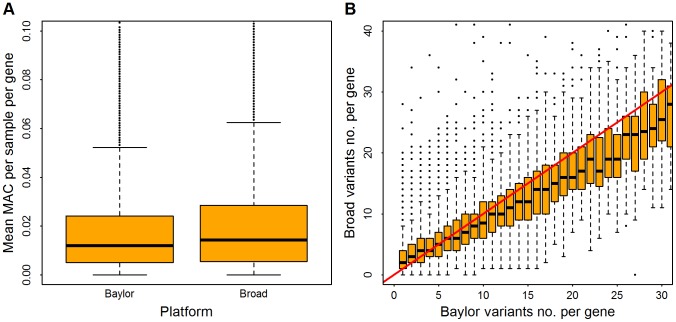
Distribution of rare variants per gene in Baylor and Broad data sets after filtering. Minor allele counts (MAC) are restricted to variants with minor allele frequency 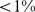. Panel (A), distribution of mean MAC per sample, averaged over all genes. Panel (B), in the Baylor samples, genes were binned based on the counts of rare variants (which range from 1 to 30); for each bin the vertical axis shows the distribution of counts (boxplot) from the same genes in the Broad samples. The red line indicates an equal count in Broad and Baylor.

Post filtering, the Broad and Baylor data sets had similar numbers of minor allele calls per sample per gene (Figure 1A). The Baylor variant count was slightly greater than the Broad count (Figure 1B), due in part to the larger number of samples in the Baylor data set. The average count of rare variants per gene was 9.24 for Baylor and 8.82 for Broad. Association analysis was limited to non-synonymous variants that had minor allele frequency (MAF) less than 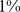. A total of 156,636 and 152,851 variants were retained in the Baylor and Broad samples, respectively. After filtering 9,738 and 5,808 indels were retained in the Baylor and Broad samples, respectively.

### Meta- Versus Mega-Analysis

Information from two or more datasets can be combined via meta-analysis with the weighted Z-score approach [32]. In the context of the SKAT test this approach assimilates gene-level information without consideration of the directionality of any single variant effects. Alternatively, if the data are combined after careful filtering and harmonization, it is possible to analyze all data simultaneously using a mega-analysis approach.

**Figure 2:**
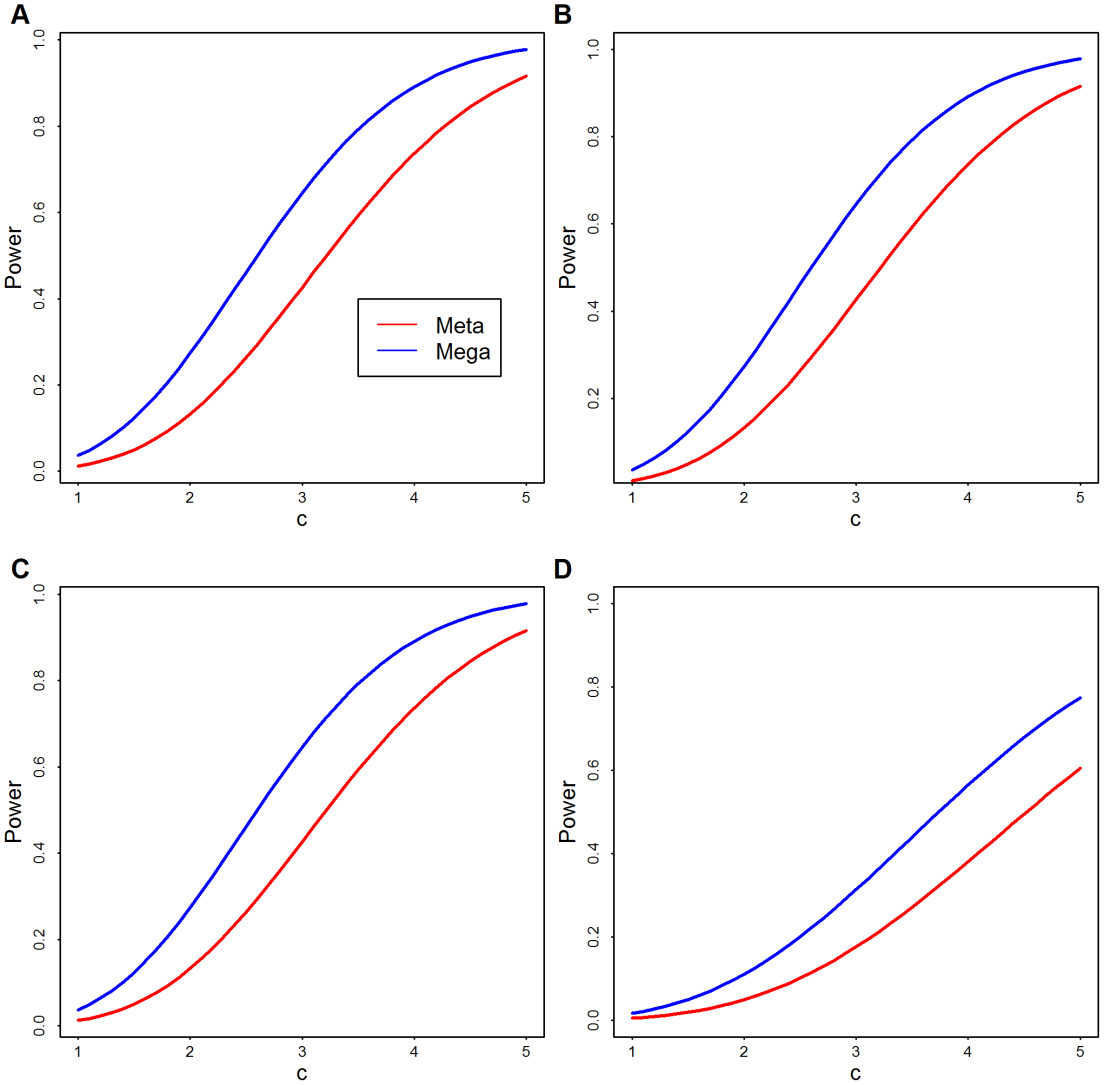
Theoretical power comparison: Meta versus Mega. Theoretical power functions of meta- (red) and mega-analysis (blue) at significance level of 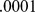. 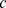 is the strength of signal per variant and 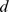 is the number of rare variants. (A) 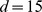; (B) 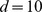; (C) 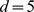; and (D) 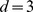.

For a theoretical comparison of these approaches, see the Methods; here we provide empirical analysis. To compare analytically the power of meta- and mega-analysis we assume two data sets have the same sample size and rare variants at the same locations. Results of this analysis show that, regardless of the number of variants, mega-analysis has greater power than meta-analysis, unless the signal is so strong that both have power close to one (Figure 2).

**Figure 3:**
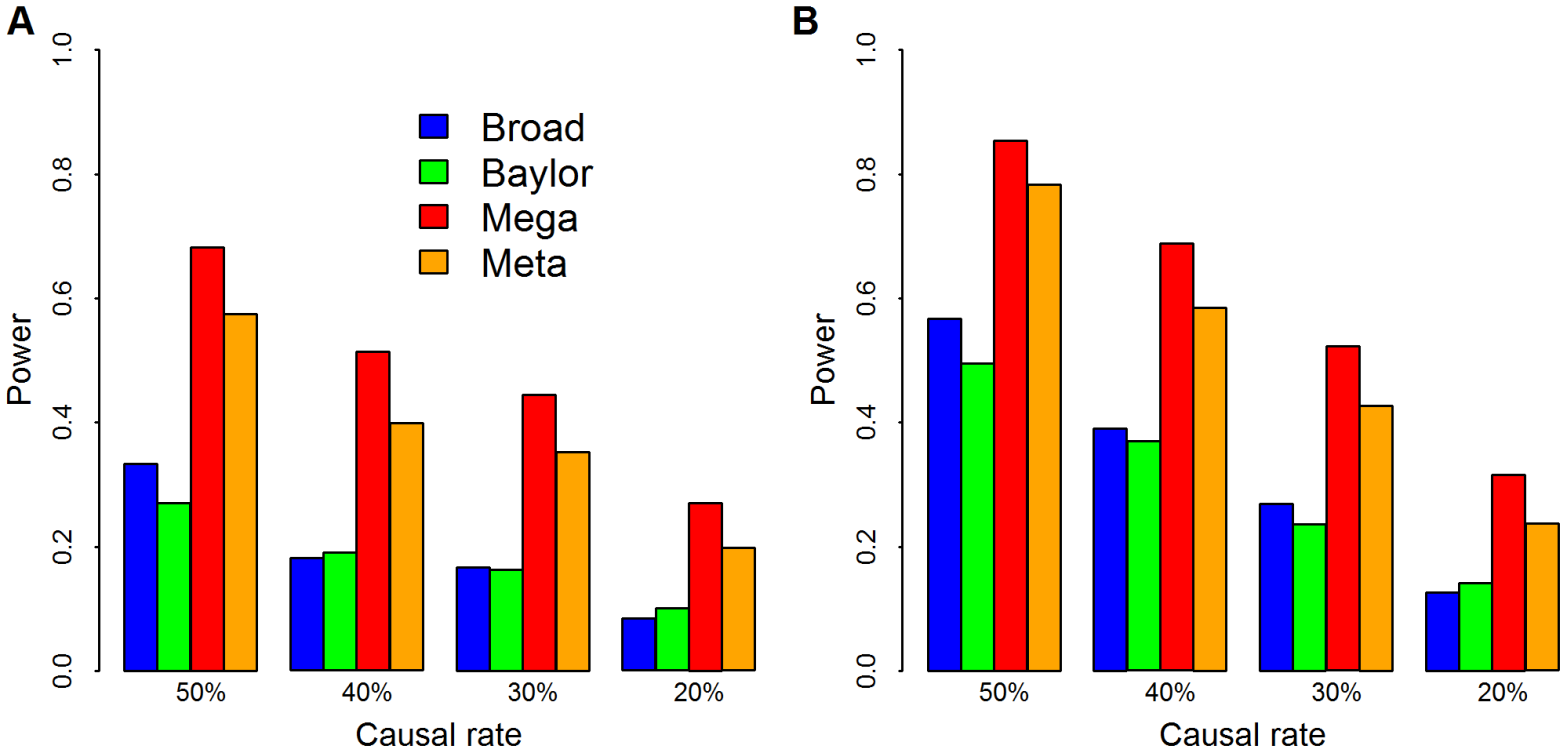
Simulation of power. The empirical power comparisons of SKAT applied to Broad (blue), Baylor (green), and combined via mega- (red) and meta-analysis (orange). We use causal variants to generate the phenotype based on the model in Eqn. 1 with 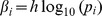. Causal rate is the fraction of variants with 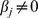, which varied from 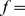
20% to 50%. We choose weights 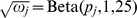 and use SKAT to calculate the p-values for Baylor, Broad and merged data sets. We combine all singleton variants as a super-variant. For meta analysis, the weighted Z-score method combines the two p-values from Baylor and Broad for each gene. Panel (A) 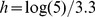 and the significance level is set at .001; in panel (B) 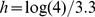 and the significance level is set at .01.

More realistic power comparisons can be made based on the observed Baylor and Broad variant calls directly in simulation. We focus on the 1090 genes with the largest number of variants to obtain the greatest flexibility for configurations of causal variants. From the combined list of variants, some of which are observed only in Baylor or Broad, but not both, and some of which are shared, we randomly pick a fraction 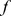 as causal variants. We use causal variants to generate the phenotype based on the model in Eqn. 1 with odds ratio inversely proportional to allele frequency. The fraction of rare variants that are causal varies from 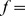
20% to 50%. In the analysis we upweight variants inversely proportional to allele frequency using SKAT's default setting. We also use SKAT to calculate the p-values for Baylor, Broad and the merged data sets based on its standard approximation technique. For this simulation analysis and for all our other data analysis, we combine all singleton variants as a super-variant. For meta-analysis the weighted Z-score method combines the two p-values from Baylor and Broad for each gene. Notice that in this analysis, mega-analysis performs better than meta-analysis under a variety of different distributions of causal variants and different log odds ratios (Figure 3).

To gain intuition into the comparison between meta- and mega-analysis, consider combining information across two dataset of approximately equal size. If, in the combined sample and for a particular variant, we observe all of the rare alleles in cases and none in controls, then the evidence for association is higher than if we combine statistics in which half of the rare alleles are observed in cases from each of two sub-samples. For example, for a variant observed 4 times, twice in cases from both subsamples, the mega and meta p-values are .06 versus .17, respectively. The difference in evidence occurs because there are five ways 4 alleles can be partitioned between cases and controls in the mega dataset (4∶0, 3∶1, 2∶2, 1∶3 and 0∶4); however, there are only three ways that 2 alleles can be partitioned between cases and controls. Thus with a larger sample, it is possible for rare alleles to obtain more unusual configurations. As variants become extremely rare the situation becomes more unfavorable to meta-analysis. Unless the sample is very large, most samples will draw only one copy of the rare allele and in this scenario neither of the two case-control configurations is unusual. With singleton variants SKAT can only gain information about association if the rare variants are grouped to form a super-allele.

Alternatively, mega-analysis also has advantages when considering rare alleles with no effect. If, for a particular variant, we observe half of the rare alleles in cases and half in controls in the combined sample, but all of the alleles are in cases in the first sample and all are in controls in the second sample, then the evidence for association is appropriately diminished by considering the full sample simultaneously (for 6 variants, mega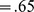 versus meta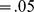). If there were only one variant per gene, it would be possible to adjust the meta-analysis to capture the sign of the association and overcome this weaknesses; however, gene-based statistics rely on having multiple variants per gene to gain power. With multiple variants, the power differential in mega versus meta occurs because mega-analysis assimilates information variant by variant, cancelling out false signals that differ in direction of association across data sets and capitalizing on true signals that match in direction. By construction, meta-analysis is restricted to combining information at the gene level post hoc, rather than at the variant level. In total, these comparisons explain why mega-analysis has greater power than meta-analysis for statistical tests such as C-alpha and SKAT, that are based on the distribution of rare variants across cases and controls.

### Distribution of AASC Data

**Figure 4:**
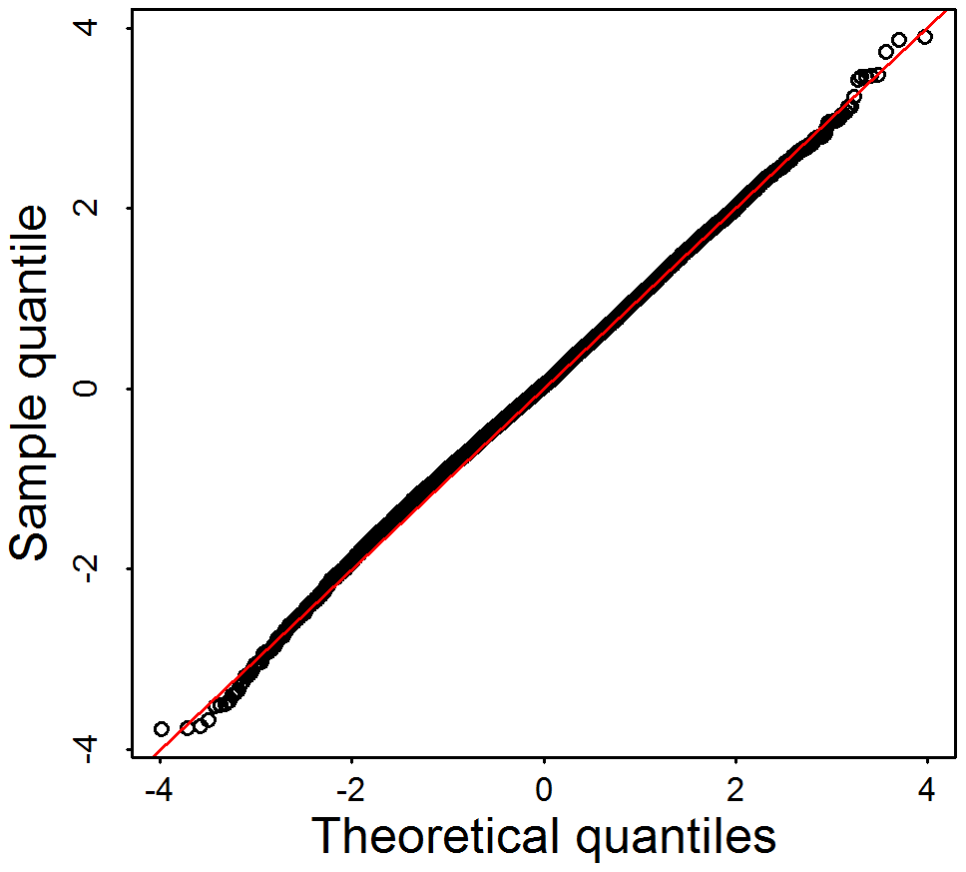
Q–Q plot of simulation tests under the assumption that linkage disequilibrium among rare variants has little impact on the distribution of the test statistic. 144 genes are selected from the Broad data set. Each gene has exactly 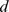 rare variants, 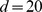. For each gene, we first randomly assign the phenotypes for 913 samples based on a coin toss, then calculate the test statistics 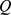, and corresponding p-value computed under the assumption that 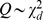. We repeat this 100 times per gene, to obtain more than 10,000 p-values.

To evaluate how sensitive the test statistic is to linkage disequilibrium typical of rare variants, we select 144 genes that have exactly d = 20 variants in the Broad data set. Using these data we randomly assigned case-control status to generate a null distribution for test statistics. With no linkage disequilibrium structure among rare variants, and appropriately chosen weights, the score test statistics 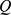 is known to follow a 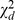 distributions under the null hypothesis. Alternatively, notable dependencies among rare variants result in a statistic that follows a mixture of 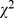 distributions, with degrees of freedom less than 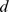. Results from simulations under the null in the form of a Q-Q plot (Figure 4), show that the independence assumption is a reasonable approximation for these data.

**Figure 5:**
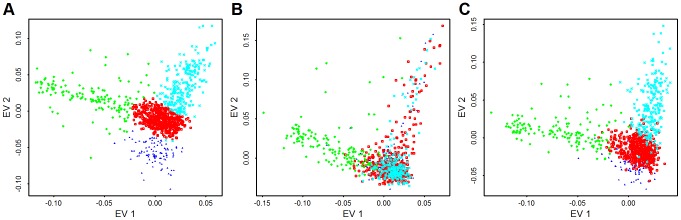
PCA from common variants, low frequency variants, and both types of variants. Plotted are the first eigen-vector versus second eigen-vector for Broad samples. Eigen-vectors are obtained by applying PCA to all common variants that have no missingness (56,607 variants) (A), all low frequency variants that have no missingness (29,509 variants) (B), and both type of variants (C). The colors are obtained by clustering individuals based on their coordinates in panel (A) using model based clustering [51].

For association analysis of common variants (CVs, MAF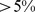) it is common practice to control for ancestry by regressing out the most predictive eigen-vectors for ancestry derived from a representative sample of CVs [29]. To determine if the distribution of rare variants varied in ancestry space similarly to CVs, we plot individuals based on their ancestry coordinates [33] using three sets of single nucleotide variants (SNVs): CVs, low frequency variants (LFVs, 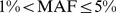), and both types of variants (CVs+LFVs). The ancestry coordinates are the eigen-vectors obtained by applying principle components analysis to CVs (14,702 CVs used in Baylor and 56,607 CVs used in Broad), LFVs (8783 LFVs used in Baylor and 29,509 LFVs used in Broad) and CVs+LFVs respectively. The variants used for PCA have no missing genotypes. We find that individuals cluster fairly similarly for CVs versus LFVs in eigen-vector 1, but less so for eigen-vector 2; and individuals cluster almost identically for CVs and CVs+LFVs (Figure 5 for Broad and Figure S1 for Baylor; notice that the similarity of clusters observed in CVs is apparent using EVs 1 and 3 for CVs+LFs). In the subsequent data analysis we explore the effect of using eigen-vectors from CVs and LFVs to control for confounding due to population structure.

**Figure 6:**
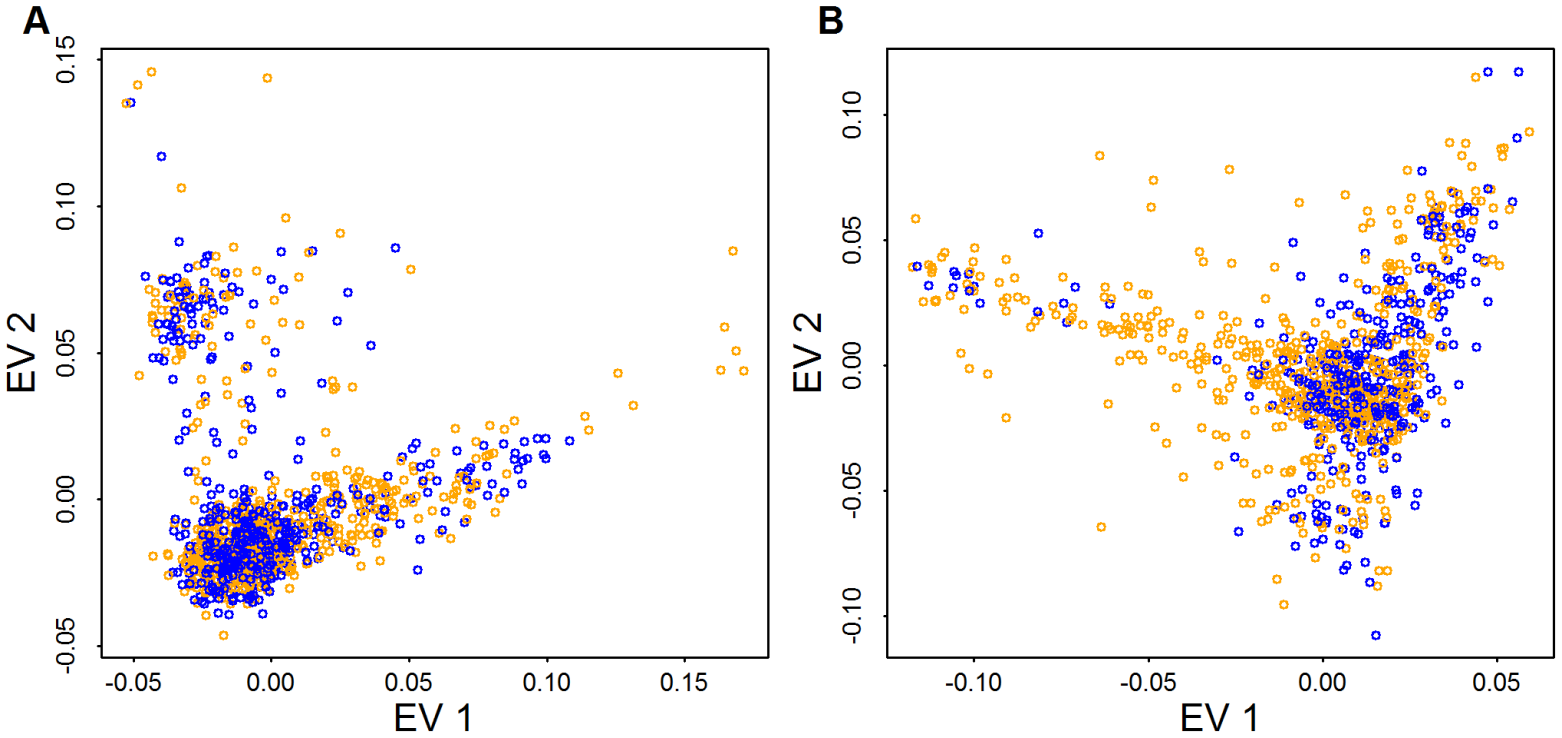
PCA for case (orange) and control (blue) samples. Panels (A) and (B) plot the top two eigen-vectors for Baylor and Broad, respectively. Eigen-vectors are obtained by applying PCA to all common variants (CVs) that have no missingness (14,702 CVs used in Baylor and 56,607 CVs used in Broad).

Cases and controls included in the AASC sample have been chosen to have matching ancestry based on eigen-vectors derived from CVs obtained from GWAS genotyping platforms [10]. Examining the distribution of cases (orange) and controls (blue) from Baylor and Broad plotted versus the top 2 eigen-vectors calculated from CVs in the exome shows that the samples are fairly evenly distributed in ancestry space but many of the subjects on the boundary of the eigenspace are cases (Figure 6). When combining Baylor and Broad samples into a common eigen-space, it is evident that the two samples overlap substantially (Figure S2). The Baylor sample, however, includes greater diversity.

**Figure 7:**
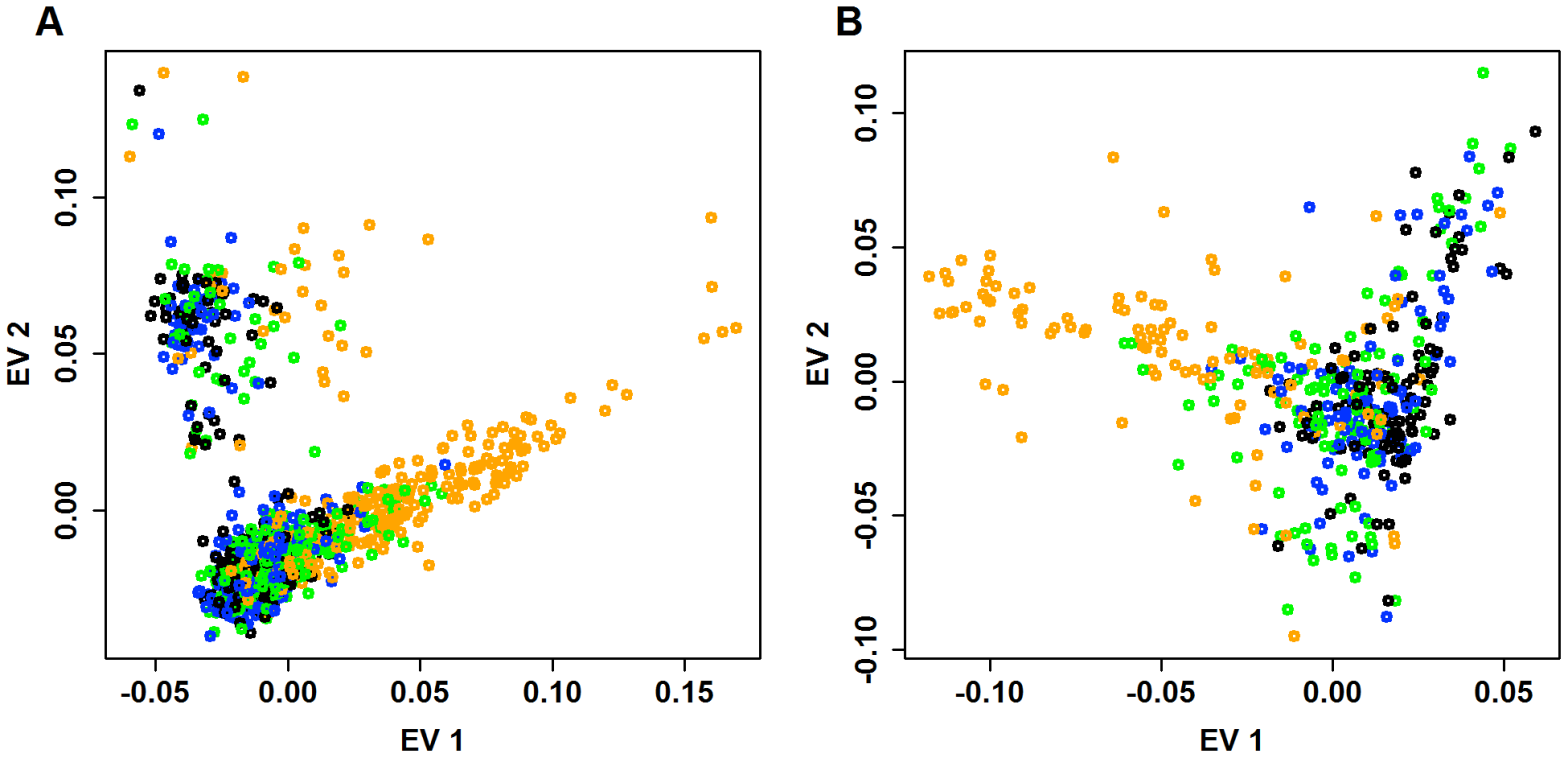
Distribution of doubletons as a function of the eigen-map. The first eigen-vector versus second eigen-vector for (A) Baylor and (B) Broad samples. Eigen-vectors are obtained by applying PCA to all common variants. For each individual, we count the number of doubletons. To indicate the relative number of doubletons per individual, points are color-coded as follows: black (bottom 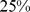: fewest doubletons), blue (next 25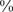), green (next 25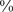), and orange (top 25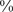: most doubletons) within the Baylor and Broad samples, respectively.

As a first step to investigate the distribution of rare variants, we identify all pairs of individuals who share doubleton variants, i.e., each had one copy of an SNV seen only twice in the entire sample. Doubletons are of interest because they are the rarest variants in our sample for which we have strong confidence in the variant calls. When we tally the total number of doubleton variants possessed by each individual in the Baylor case sample, the distribution of the doubleton-count varies widely, with some individuals having a far greater share of these rare variants than expected due to chance. We examine the distribution of doubletons as a function of the eigen-map. Figure 7 displays the relative count of doubletons in the 2-dimensional eigen-map for the Baylor and Broad samples. Individuals with the largest number of doubletons tend to be clearly separated from the majority of the subjects in ancestry space by the top two eigen-vectors.

**Figure 8:**
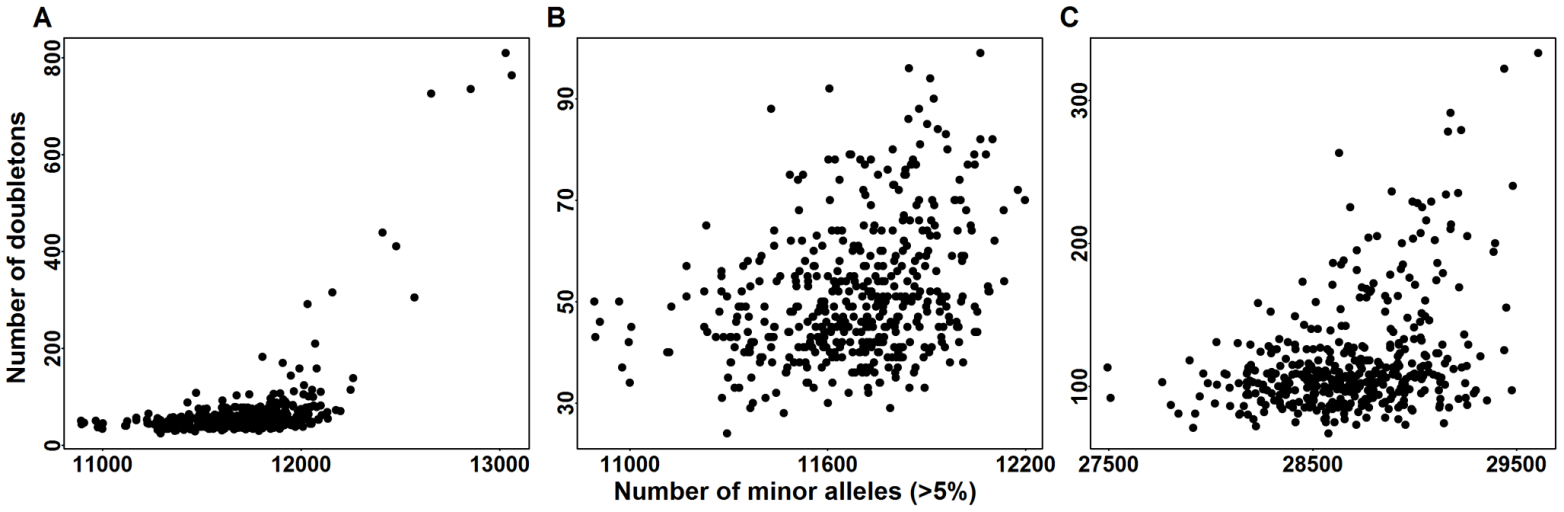
Doubletons counts versus minor allele counts (MAC_c) in common variants (CVs). MAC_c are computed for all variants with minor allele frequency 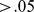. Panel (A) is the doubleton counts of Baylor cases versus MACs of CVs in the exome. Panel (B) is a zoomed in version of panel (A). Panel (C) is the doubleton counts of Broad cases versus MAC_c of CVs in the exome.

To compare the distribution of doubleton counts with the distribution of common variants, for each individual in the Baylor case sample we tally their count of minor alleles (MAC_c) over exonic CVs. From Figure 8A, 8B it is clear that individuals with a large count of doubletons also possess a disproportionate number of minor alleles, suggesting that these individuals are toward the boundary of the European ancestry space. Indeed all of these individuals are separated in eigenspace from the majority of the individuals (Figure 7A, orange points). Furthermore, sample records suggest that many of these individuals are from Portugal, a population whose individuals have a somewhat larger component of African ancestry. The same pattern exists in the Broad case sample (Figure 7B and Figure 8C); however the Broad sample does not include any individuals with very large numbers of doubleton variants.

These findings suggest that the distribution of common variants might function as a proxy for the distribution of rare variants. Next we look to see if these descriptive analyses support the use of an eigen-map to control for confounding in rare variant tests due to ancestry. To test for association between ASD and rare variants in the AASC sample, we apply burden tests and SKAT to the filtered version of the data sets and obtain the p-values of genes in the Baylor, Broad and combined datasets. We investigate the effects of population structure by calculating the genomic control inflation factor 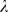 [34] when the test is performed with and without including 10 eigen-vectors for ancestry obtained from genotypes of CV [29].

Before comparing choices of eigenvectors, we investigate the behavior of the genomic control statistic, 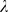, when calculated based on rare variant test statistics. SKAT has been shown to provide accurate p-values in the tail of the distribution for moderate sized samples [26]. Indeed, for these data, we also find that the nominal p-values appear to be accurate in the tail of the distribution (see below). The distribution of the p-values across the genome, however, does not follow the expected uniform distribution (Figure S3A, S3B). Specifically, for those genes clearly not associated with the phenotype (p-values 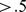) we find that SKAT tends to report p-values biased downward toward .5, causing an apparent, but uninteresting inflation in the GC factor. Notably, the algorithm for computing p-values seems to be accurate for smaller p-values; we do not find a bias in estimate of the first quantile (Figure S3A, S3B). A similar phenomenon holds true for the burden test, but to a much lesser extent (Figure S3C, S3D). This is likely due to the very small counts of rare variants. Using permutations to obtain p-values would remedy the situation, but at a substantial cost in computation.

**Figure 9:**
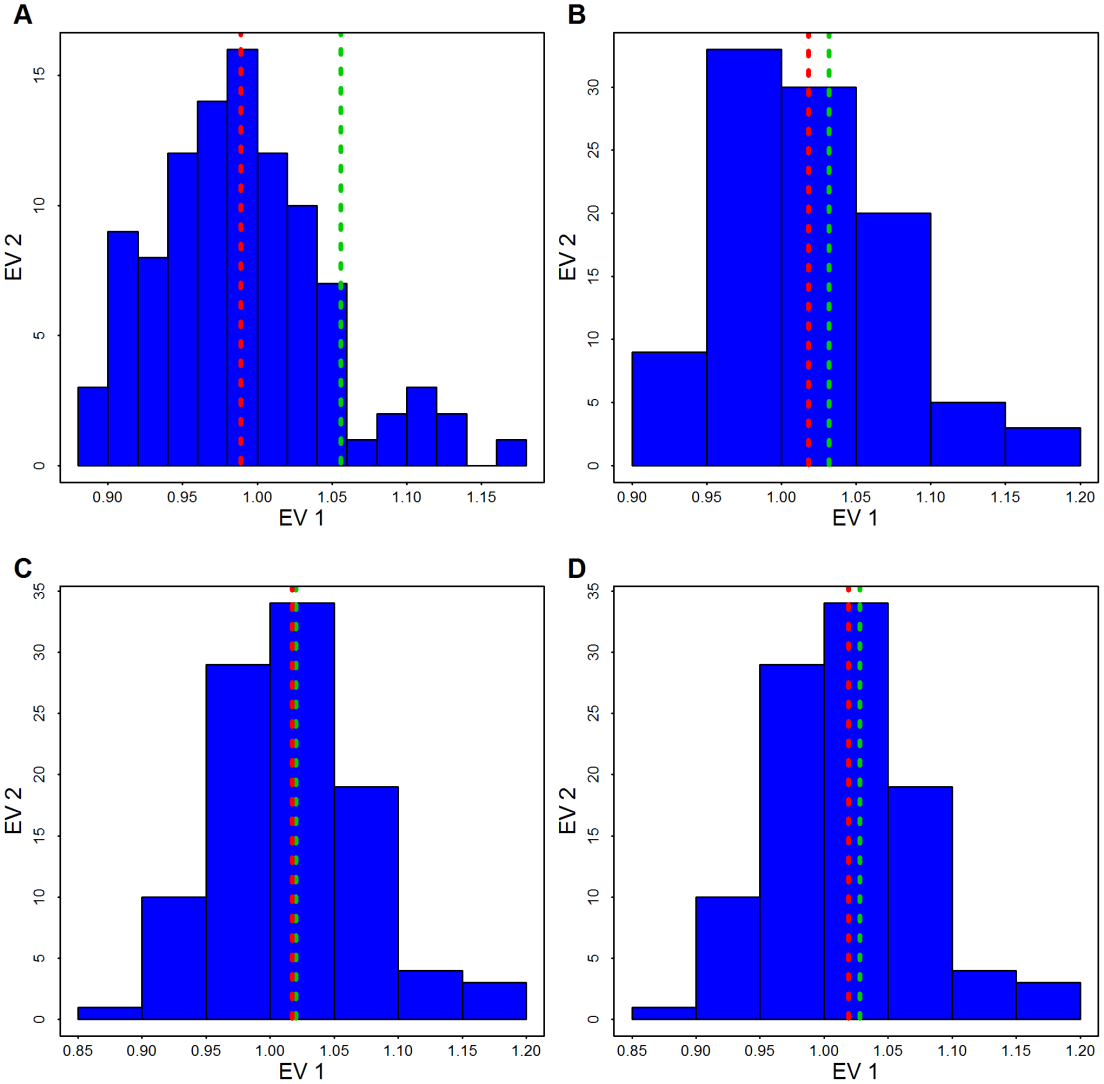
Distribution of the genomic control factor 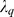. By permuting case/control status 100 times the distribution of 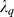 is obtained based on the 1000 largest genes. The red line shows the mean of the permutation distribution and the green line shows 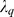 obtained from the data using (A) Broad SKAT p-values obtained without eigen-vectors; (B) Broad SKAT p-values, with common variants (CVs) eigen-vectors, (C) Broad SKAT p-values, with low frequency variants (LFVs) eigen-vectors; and (D) Broad SKAT p-values, with CVs plus LFVs eigen-vectors.

These insights into the null distribution of the rare variant test statistics lead us to calculate 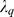, a variant on the GC principle based on the first quantile (rather than the median) of the p-value distribution. For a properly calibrated statistic 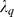 has an expected value of 1 when there is no confounding due to population structure (see Text S1). To compare the behavior of these two genomic control factors we conduct the following experiment. We calculate 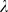 and 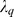 based on SKAT statistics computed for the 1000 largest genes. Then we permute case and control status 100 times, computing the genomic control factors for each permutation, to obtain the distribution of these statistics (Figure 9 and Figure S4). Notice that the observed value of 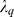 is close to the mean of the simulated distribution for all 3 choices of eigen-vectors. In contrast 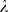 shows much greater variability and the mean of the permutation distribution is shifted further above 1, supporting our conjecture that 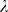 provides a positively biased estimate of the effect of confounding when using the SKAT statistic for samples like this one.

**Figure 10:**
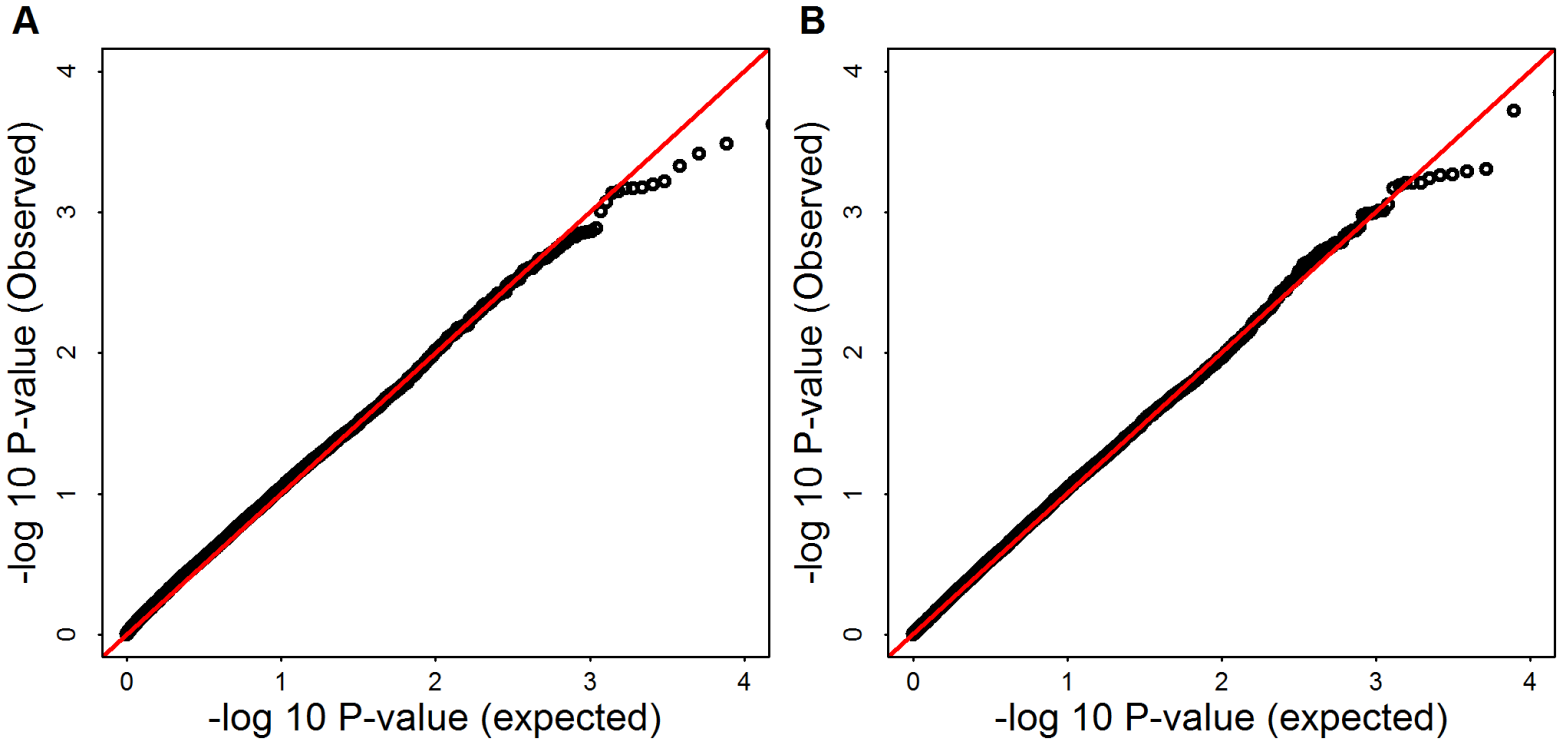
-log10(observed p-values) versus -log10(expected p-values) of SKAT and Burden test for Mega-analysis. Panel (A) shows SKAT p-values, Panel (B) shows burden test p-values. 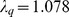 and 1.047, for mega SKAT and burden test, respectively.

Next we examine the effect of adjusting for ancestry (using CVs) on the rare variant test statistics. Notice that while 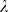 is inflated for all conditions, 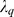 is controlled fairly well in the Baylor and Broad samples individually (Table 1); in the mega SKAT analysis there is a slight inflation (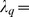
1.08). From Table 1 and from the -log10(observed p-values) versus -log10(expected p-values) plot (Figure 10) we see the distribution of the test statistics follows the null hypothesis quite closely. We conclude that adjusting for ancestry using CVs is sufficient to yield a substantial reduction in 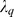.

**Table 1:** Genomic control 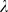 and 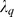 for all tests before and after PC adjustment.

	Broad		Baylor		Mega		Meta	
	no PCA	PCA	no PCA	PCA	no PCA	PCA	no PCA	PCA
SKAT 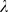	1.197	1.115	1.251	1.163	1.298	1.188	1.322	1.200
SKAT 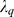	1.064	1.032	1.107	1.046	1.176	1.078	1.145	1.089
Burden 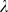	1.109	1.070	1.146	1.037	1.195	1.107	1.175	1.082
Burden 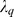	1.059	1.031	1.094	1.027	1.151	1.047	1.104	1.036

Note: These analyses are restricted to the genes that have more than 4 minor alleles in the samples used in each study. 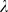 and 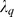 are calculated based on the median and the 1st quantile of the p-value distribution, respectively. PC adjustment is based on the common variants (CVs) eigen-vectors.

We explore this further by contrasting the results obtained when applying no correction versus correction based on eigen-vectors derived from CVs, LFVs and CVs+LFVs and find that the corrected results are nearly indistinguishable regardless of the scenario (both data sets individually, SKAT or burden test, meta- or mega-analysis; Table S2). For example, in the Broad sample and the SKAT statistic, using no eigenvectors yields 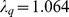 compared to 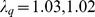, and 1.03, derived using CVs, LFVs and CVs+LFVs, respectively.

### Association Analysis of AASC Data

As described previously most analyses of the data use Filter DpBal to screen called variants. Because one should always be concerned about the possibility of screening out risk variants by this filtering process, we first examine the number of genes exceeding a threshold (i.e. signals) for 3 filters ranging from lenient (Filter PASS) to stringent (Filter DpBal; Table 2). Applying the test statistic to the individual data sets we find no large excess of signals even for the most lenient filter. However, for mega-analysis, filtering is essential to avoid false positive signals. Consider the number of genes with p-values less than .001; with baseline filtering (PASS) we observe a significant excess of such genes (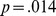), but no excess with any other filters (Table 2). Next, considering the number of genes with p-values less than .01 the pattern continues; with baseline filtering (PASS) we observe a highly significant excess of such genes (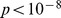), but this large excess is absent for Filter DpBal (Table 2). It is quite likely that the slight excess of genes with p-values less than .01 after filtering is due to real, but weak signals in a small set of genes. A candidate diagnostic for filtering is matching of minor allele count per person of rare variants (MAC) across platforms (Table 2). However total MAC is a crude measure of alignment. Diagnostic plots such as Figure 1B give a more insightful comparison across genes and we conjecture that a filter chosen to attain good alignment of MAC across genes is a candidate for successful data harmonization. MAC should also be similar across cases and controls for most genes; for Filter DpBal, MAC per person is 330 and 300 in cases and controls, respectively.

**Table 2:** Number of significant genes (and expected number) under different filters.

	Baylor		Broad		Mega		MAC	
Filter/ 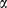	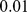	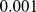	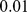	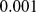	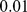	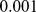	Ba	Br
Filter PASS	97(97)	7(10)	77(78)	10(8)	195(127)	22(13)	462	391
Filter MISS	79(73)	7(7)	77(76)	10(8)	133(113)	11(11)	338	379
Filter DpBal	67(66)	6(7)	69(70)	11(7)	123(106)	11(11)	305	351

Note: These analyses are restricted to the genes that have more than 15 minor alleles in the samples used in each study. MAC columns show the number of minor alleles called per sample, Ba: Baylor, Br: Broad. Filter PASS includes all variants that score a “Pass” based on GATK, Filter MISS: missingness 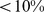, Filter DpBal: missingness 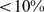, depth 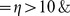 balance 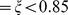 for Baylor, 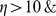 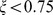 for Broad.

While filtering is beneficial to remove false positives, it has the potential to remove real signals as well. We explore the effect of filtering on a particular gene (*SCN2A*) that has been demonstrated to be an ASD gene based on 3 recurrent *de novo* loss of function mutations [4], [5]. In the Baylor sample, with Filter PASS we obtain a suggestive p-value of .009, but many of the observed variants have high missingness, very low depth and poor balance of alleles. With Filter MISS the p-value is .033. Finally, with additional filtering the signal is removed altogether. (Specifically, Filter DpBal removes 2 putative severe missense mutations [35] and 1 putative loss of function variant from cases.) There is no evidence of association in the Broad sample for this gene.

Prior to filtering, a sizable fraction of the loci in which a variant is called for one subject cannot be called – either heterozygous or homozygous – for other subjects; it is current practice to remove loci that have variant calls for some subjects, but 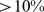 of subjects have missing calls. After filtering (Filter DpBal), .3% of the values are missing, but the missingness is not evenly distributed across sites or case/control status (Table 3). Most notably this “missingness rate” in Baylor cases is twice as high as the missingness in Baylor controls and 90% of the missingness arises from the Baylor site. Although differential missingness has the potential to cause false positive associations, differences between cases and controls within each data set are not so high as to induce an excess of false positive associations in meta-analysis even in the unfiltered data; however, if we apply mega-analysis to the unfiltered data, we obtain a significant excess of genes with p-values 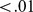 (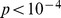; Table 4). This problem is remedied by applying Filter DpBal: after filtering, which removes loci with high rates of missingness, we obtain no excess of small p-values for the SKAT mega-analysis test statistics. When evaluating this issue at a finer scale after filtering by looking at the effect of differential missingness at the gene level, we find no association between the test statistic and differential missingness (Figure S5).

**Table 3:** Counts of missingness per sample after filtering.

	Baylor		Broad	
	case	control	case	control
Missing	1,104	561	92	117
Not Missing	124,459	125,002	170,165	170,140

Note: These analyses are for all non-synonymous variants with MAF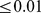.

**Table 4:** Number of nominally significant genes before and after filtering.

	Meta		Mega	
	Observed	Expected	Observed	Expected
Filter PASS (MAC 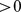 )	156	168	219	168
Filter DpBal (MAC 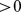 )	132	156	156	156
Filter PASS (MAC 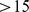 )	133	127	195	127
Filter DpBal (MAC 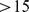 )	96	106	123	106

Note: Significance level is 0.01, not corrected for muliple testing. The analyses of the first two rows are for all genes that have at least one MAC in Baylor and Broad dataset. The last rows are restricted to the genes that have more than 15 minor alleles after combining Baylor and Broad datasets.

Neither SKAT nor burden gene-based tests produce a test statistic exceeding the threshold for exome-wide significance (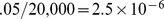). Genes with p-values 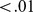 are reported in Table S3. Note that nearly half of these genes have more rare variants in controls than cases, suggesting a protective effect, but we view this as unlikely. Moreover, the evidence is also not sufficiently compelling to replicate any known ASD gene. To explore this last issue in more detail we compile a list of genes with at least two functional *de novo* mutations identified in the recent ASD studies [4]–[6], [8] (Table S4), and we examine the 114 ASD genes cited by [36] as ASD genes (Table S5). For all genes in these lists we obtain the p-values of SKAT and the burden tests applied to Broad and Baylor samples separately and jointly by mega-analysis. None of the genes yield compelling signals, arguing strongly that our power is insufficient to detect associations with rare variants without further information to guide our analysis.

## Discussion

Studies of the distribution of *de novo* copy number and sequence variants in ASD and control subjects invariably find elevated rates of damaging de novo events in ASD subjects [1]–[8]. These studies also invariably find relatively little convergence of *de novo* events on particular loci in the human genome. These results are consistent with only one conclusion about the genetic architecture of ASD, namely that there are hundreds of genes in the genome that can affect liability, possibly more. Indeed various statistical analyses of the data support this conclusion [5], [8].

Another common theme of ASD studies is that while *de novo* events are rare, they can successfully identify ASD liability genes, and in general the distribution of rare variation has been a key tool for gene discovery [37]. By contrast common variation has not yet proven an effective tool for discovering replicable ASD genes, although there are tantalizing findings [10].

With these observations in mind the AASC has implemented a study of rare variation in ASD based on WES [38]. Here we report on data from almost 2000 ASD subjects and controls. We find the distribution of rare variation between cases and controls is remarkably similar, showing that ASD risk genes cannot be identified in a case-control sample of this size. Indeed, even known ASD genes showed little association in this study. This finding is in keeping with other studies of rare variants, but with quite different phenotypes, supporting the conjecture that rare variant association studies require large samples [19], [39], [40]. With respect to the genetics of ASD, the results are also consistent with the inference from *de novo* studies that there must be hundreds genes affecting liability to ASD [3]–[6], [8]. These results underscore the scale of the challenges ahead in our effort to discover ASD genes. Large samples must be amassed and assessed and effective study designs implemented [41].

To gain insight into the limited power of this study, consider three scenarios: (A) the gene has 15 variants, each with MAF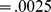, for which all have odds ratio of 4; (B) the gene has 20 variants, each with MAF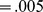, for which 10 have odds ratio of 3; and (C) the gene has 40 variants, each with MAF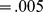, for which 30 have odds ratio of 2. We list the required samples size of each scenario in Table S6 to achieve a power of 50% and 80% per gene (with a p-value threshold of 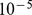). Even though the power of mega-analysis is only 0.31, 0.11 and 0.06 for our study, assuming these scenarios were realistic, power would have been sufficient to discover a fraction of the large number of ASD genes present in the genome. We conclude that these scenarios do not describe likely models for risk genes in ASD.

As with GWAS, to assimilate large samples and gain power, multiple studies must be combined. In the analysis of samples from multiple studies, meta-analysis, based on Z-scores, has become the norm for most genetic investigations. This form of meta-analysis has power equal to mega-analysis for single variant tests [42], hence it is reasonable to assume that meta-analysis is generally superior to mega-analysis because the former more easily accommodates heterogeneity across studies. A notable result from our study is that these results do not carry over to gene-based tests such as SKAT. In that setting mega-analysis has considerably more power than meta-analysis because mega-analysis assesses the concordance of association for a variant across all sites and then combines information across all variants within a gene. In this way, the method separates true signals from false ones and attains a greater signal to noise ratio. In contrast, meta-analysis combines information across studies at the gene level and hence can not assess the pattern of signals at the variant level across sites.

A drawback of mega-analysis is that we encounter challenges when combining datasets collected across multiple studies, which can differ in many respects due to the use of different sequencing platforms and protocols. For instance, these differences lead to differential coverage by exon and different alignment errors. Even the best laboratory process has measurement error and these errors are exacerbated when they differ across batches of samples, particularly if they differ between cases and controls. For these reasons caution must be exercised if one is to reap the benefits of mega-analysis. Indeed, even after careful filtering, heterogeneity between sites could account for the modest inflation in the associate test statistics and the genomic control factor after combining sites via meta- and mega-analysis.

In this study we construct extra filters to ensure that the distribution of rare variation of the WES data is similar for the two centers. We find good results filtering called variants by fraction of missing data, read depth, and balance of alternative to reference reads. Ideally a filter is tuned by measuring some individuals on multiple platforms. We tune our filters using subjects measured twice. If such data are unavailable, however, we find that another promising approach is to compare minor allele counts (based on rare variants) per gene. A good filter is one that aims to equilibrate these quantities.

Even with the most minimal filtering we observe no excess of positive signals for association within the individual data sets, but for mega-analysis we observe a great number of positive associations. These false discoveries are diminished, however, after filtering. Likewise mega-analysis is more susceptible than meta-analysis to the impact of differential missingness across platforms and across case/control status. Indeed, without filtering, mega-analysis has many false discoveries but meta-analysis did not. However, using filtered data we find that mega-analysis is quite robust to differences in missingness rates across platforms and case/control status, although we recognize that this robustness could fail for more extreme heterogeneity of missingness. Still our study has some differences in missingness and yet does not produce detectable false discoveries. From our analyses we conjecture that filtering that removes variants with 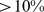 missingness (per data set) is largely effective.

When combining data sets the effects of population substructure on association is also a concern due to clustering of rare variants in ancestry space [40], [43]. Even though our case-control samples are approximately pair-matched by ancestry in the study design, we find weak evidence of population structure confounding the test of association. In our data these effects could be mitigated by regressing out principal components of ancestry using common variants or low frequency variants. This result supports findings of [44], but is contrary to other predictions [43]. Thus, although rare variants tend to be younger, and therefore distinctly clustered in populations, in our sample estimates of ancestry derived from common variants capture the major features of the distribution of rare variants in ancestry space.

In conclusion we find that WES data on nearly 2000 samples collected for a case-control study are insufficient to discover novel liability genes for ASD, even after applying efficient methods like mega-analysis and controlling for ancestry effectively. These results demonstrate that much larger samples will be required for effective gene discovery and lend further support to the prediction that there are hundreds of genes that impact ASD liability in the human genome.

## Methods

### Data

The AASC whole-exome sequencing data includes 1039 independent subjects diagnosed with autism spectrum disorders (ASD). Subjects were selected to be of European ancestry, based on genetic (eigen-vector) analysis and European origin. Samples were selected from the Autism Genetic Resource Exchange (AGRE, research.agree.org), the Autism Simplex Collection (TASC [45]), National Database for Autism Research (NDAR, ndar.nih.gov) and the Boston's Autism Consortium (autism.consortium.org). 870 independent controls were selected from the NIMH repository (www.nimhgenetics.org) to be of similar ancestry to cases (Baylor cases: 440 males, 65 females; Baylor controls: 240 males, 251 females: Broad cases: 429 males, 105 females, largely from the autism Consortium; Broad controls: 177 males, 202 females.) The Broad cases included probands only from trios. These trios were previously analyzed for *de novo* variants [5]. *De novo* variants were included in these analysis.

To evaluate sequence quality, 7 controls were sequenced at both centers. The capture/enrichment assays used were Nimblegen (Baylor) and Agilent (Broad). The Baylor samples were sequenced using the Solid platform and called with AtlasSNP 2 [30]. The Broad samples were sequenced using the Illumina platform and called with GATK [31]. Standard filters were used as part of both pipelines to produce calls for SNVs and indels. For details see Text S1.

In general, the MAF of SNVs matched well for the majority of the SNVs in the two data sets, but some differed considerably (Figure S6). One source of differences was the read depth: Broad reads had greater mean depth and also greater variability than Baylor reads (Figure S7). Overall counts of variants differed by platform (Table 5). We utilized additional filters to make these data sets more compatible. Relying on the validated *de novo* variants [5] and 7 overlapping samples we constructed an additional 3-round filter (see Text S1 and Table S7). First, for each data set, we excluded the variants that had 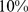 or more missing calls. Second, we discarded the variants that had average depth less than 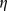. Third, we filtered the variants by the quality of the minor allele call. We defined the balance of depth for each minor allele call as the reference depth divided by the total depth. If more than half of the minor allele calls had a balance larger than 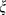 or depth smaller than 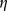, we discarded this variant. Based on these features we constructed 6 filters denoted by PASS, MISS, DpBal, B, C and D of increasing stringency. For Filter DpBal, 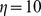, 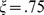 for the Broad data set and 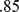 for the Baylor data set; for Filter B, 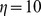, 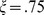; for Filter C, 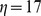, 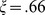 for the Broad data set and 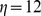, 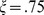 for the Baylor data set; for Filter D, 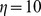, 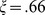. If less than half of the minor allele calls had a balance larger than 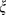, we kept this variant but changed the specific calls that did not pass the quality threshold from heterozygote to the common homozygote call.

**Table 5:** Counts of non-synonymous variants in Baylor and Broad before filtering.

	Single	Double	RVs	LFVs	CVs	total
Baylor	193,281	22,355	29,363	9800	14,159	268,958
Broad	119,648	17,628	27,644	9996	16,327	191,243

Note: Single: count of singletons; Double: count of doubletons; RVs: count of variants with MAF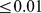 and not singletons or doubletons; LFVs: count of variants with MAF 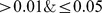; CVs: count of variants with MAF 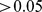.

Two rounds of filtering were performed on called indels. First, for each data set, we excluded indels with MAF greater than 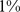 or more than 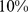 missing calls. Second, we excluded indels that had more than six calls in one data set and none in the other data set.

### Statistical Analysis

For 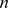 subjects sequenced, let 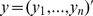 denote the vector of phenotypes. For a gene with 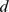 rare variants let 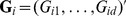 be the 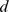-dimensional genotype vector. For dichotomous phenotypes we consider a logistic model:
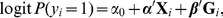
(1)where 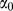 is the intercept, 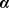 is a vector of regression coefficients for fixed covariates 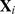 such as sex and ancestry, and 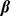 is the vector of log odds ratios for the genetic variants. For analytical purposes only we also discuss the corresponding linear model for continuous phenotypes:
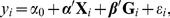
(2)where 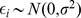. Without loss of generality, we assume 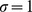.

We want to test the null hypothesis 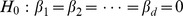. One way to increase the power of the test is to assume that 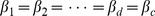 and test if 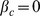 [46]. Tests of this hypothesis are often called burden tests. To add prior information to this test, the weighted sum test has been proposed [22]. The idea of weighted sum test is to use 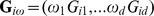 rather than 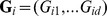 in model (1) so that biologically more plausible risk variants have larger weights in the test statistic. In our study, we use the weighted sum test with weights 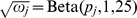, where 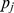 is the MAF of 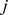
th variant. To implement the test, the genotypes 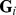 in model (Eqn. 1) are replaced by a single composite term 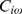, which is the weighted sum of the genotype values of all rare variants 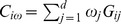. To assess significance of 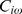 as a predictor, we use the score test.

There are drawbacks to a burden test. It assumes that all rare variants in the gene have the same direction and magnitude of association. In reality, variants can be damaging, protective, or have no effect, potentially reducing the power of the test. To overcome these drawbacks, the C-alpha test [25] has been proposed. The test is sensitive to unusual patterns in the distribution of rare variants across cases and controls. It has good power if most of the copies of a rare variant occur in cases (or controls), yet unlike the burden test, this pattern can vary across SNVs. SKAT [26] is a generalization of the C-alpha test. It has the advantage of readily incorporating covariates, but without covariates it reduces to the same form as C-alpha. This statistic is based on the generalized linear model (Eqn. 1 or 2), with random effects for the 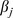
's, which are assumed to follow an arbitrary distribution with mean zero and variance 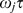 [47]. The test statistic is the score test for 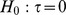, which is of the form
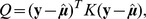
where K = GWG' is the kernel matrix, 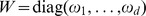 is a weight matrix, and 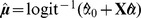 for the logistic model (1) and 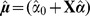 for the linear model (2). The SKAT statistic can also be expressed in terms of the individual score tests for evaluating 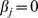 for each of the 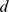 variants; let 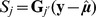, 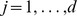, then
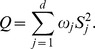
The null distribution of 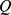 is approximately a linear combination of 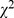 distributions,
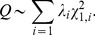
(3)The SKAT p-values can be obtained by applying Davies exact method [48] to the data and inverting the characteristic function of 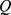.

### Meta- Versus Mega-Analysis

Suppose we have samples from two (or more) datasets. To fix ideas, consider two data sets, 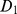 and 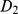 where 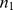 and 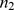 are the sample sizes, respectively. To perform meta-analysis using the weighted Z-score approach, first compute 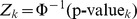, where the p-values are obtained for each data set 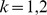 independently, and 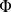 is the standard normal distribution function. Then the meta-analysis p-value is computed from 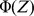, where
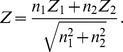
When applied to the SKAT test, this statistic combines information at the gene level without consideration of the directionality of any single variant effects.

We formally consider the SKAT test statistics in meta- and mega-analysis by deriving a closed form expression for the power of meta- and mega-analysis under restricted conditions. In the Results we show via simulations that the results hold more generally. Analysis is greatly simplified by choosing weights 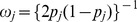, a choice suggested in [22]. This weight is equivalent to scaling 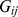 as
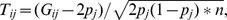
where 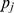 is the MAF of the 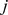
th variant. For the following calculations we also assume no linkage disequilibrium (LD) between rare variants [49], [50]. Consequently we have
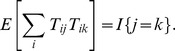
In the Results we show that this assumption appears to be reasonable in the AASC data. Under these conditions and assuming there are no covariates, we note that 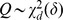, with
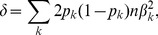
(4)for the linear model (Eqn. 2), and
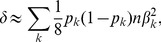
(5)for the logistic model (Eqn. 1; see Text S1).

It follows that the mega-SKAT statistic 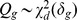, where the experiment-wise non-centrality parameter is the sum of non-centrality parameters from the individual studies: 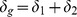. Hence, when combining 2 studies, with sample sizes 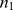 and 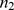, in which the 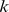
'th variant has log odds ratio 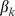, the contribution to the signal is proportional to
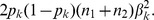
Notice that this term is approximately equal to the number of realizations of the variants in the pooled data (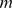 in the example above) times the square of the log odds ratio. For rare variants the number of realizations tends to be very small, emphasizing that large samples are essential to gain good power.

In a comparison of the power of meta- and mega-analysis we assume data sets 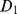 and 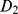 have the same sample size and rare variants at the same locations. Furthermore, building on our analysis above, we assume the individual test statistics from the two samples are distributed as 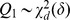 and 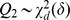. Under 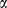-level type I error, the power function of weighted z-score meta-analysis and the power function of mega-analysis can be approximated as given in Text S1 (Eqn. S3–S4). The derived expressions are complex, but from Figure 2 we see, regardless of the degrees of freedom, mega-analysis has greater power than meta-analysis.

To gain more analytical insight, consider a gene for which each sample has sufficient coverage to detect all rare variants and that a total of 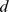 rare variants are observed. Let 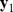 and 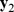 be the corresponding phenotype vectors and 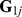 and 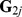 the genotype vectors for variants 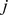, 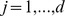. Furthermore, let 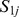 and 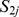 denote the 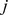
th variant scores corresponding to 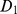 and 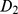. Next let's look at the test statistics for mega-analysis 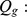


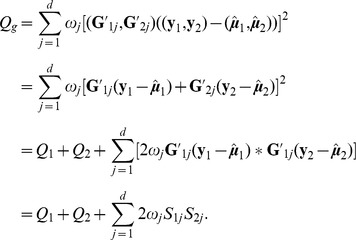
Under the alternative hypothesis, the per-variant scores 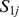 and 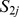 corresponding to 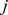
'th causal variant tend to have the same sign; positive for risk variants and negative for protective variants. Under the null hypothesis these per-variant score statistics are uncorrelated and tend to cancel each other out, on average. Consequently the final term in the expansion above tends to be positive under the alternative hypothesis and close to zero under the null.

In the Text S1 we find that the information captured by the meta-analysis statistic is approximated by the two lead terms (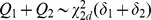). Thus this expansion reveals why mega-analysis is more powerful than meta-analysis for quadratic test statistics such as SKAT. Mega-analysis cancels out false signals that differ in sign. Meta-analysis is restricted to gene level information and hence cannot account for directionality. The strength of the signal over a gene is determined by two factors: the sum of the per-variant contributions to the signal, versus the number of degrees of freedom. Both meta and mega-analysis assimilate the same signal (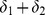), but the strength of the signal for meta-analysis is apportioned over more degrees of freedom, effectively diminishing the power. For mega-analysis, the degrees of freedom increase only if the rare variants occur at different locations in the separate studies. The power advantage of mega-analysis is most pronounced when the rare variants accumulate at common locations across data sets. meta-analysis is not able to assimilate information within a variant across data sets as efficiently.

## Supporting Information

Figure S1 PCA from common variants, low frequency variants and both type of variants for Baylor samples. Eigen-vectors are obtained by applying PCA to all common variants that have no missingness (14,702 variants) (A), all low frequency variants that have no missingness (8783 variants) (B), and both type of variants (C). The colors are obtained by clustering individuals based on their coordinates in panel (A) using model based clustering [51]. (A) and (B) are the first eigen-vector versus second eigen-vector for Baylor samples. (C) is the first eigen-vector versus second eigen-vector for Baylor samples.
(TIF)

Figure S2 PCA of Baylor and Broad samples together. first eigen-vector versus second eigen-vector for Broad and Baylor samples.
(TIF)

Figure S3 Histogram of p-values for SKAT and Burden Test. (A) and (B) are SKAT p-values for Broad and Baylor samples, respectively. (C) and (D) are Burden test p-values for Broad and Baylor samples, respectively. Green vertical lines are the 25%, 50% and 75% quantiles of p-values.
(TIF)

Figure S4Distribution of the genomic control factor 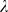. By permuting case/control status 100 times the distribution of 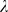 is obtained based on the 1000 largest genes. The red line shows the mean of the permutation distribution and the green line shows 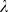 obtained from the data using (A) Broad SKAT p-values obtained without eigenvectors; (B) Broad SKAT p-values, with CVs eigenvectors, (C) Broad SKAT p-values, with LFVs eigenvectors; and (D) Broad SKAT p-values, with CVs plus LFVs eigenvectors.(TIF)

Figure S5 P-values versus Missingness. We used 5500 genes to make this plot. For each gene, we calculate the -log 10 p-values and the odds ratio of missingness in case and control. The red line is the fitted line of these 5500 observations.
(TIF)

Figure S6 MAF Comparison: Baylor versus Broad. We compare the MAF for 72,758 shared non-synonymous variants in the two data sets.
(TIF)

Figure S7 Depth Comparison: Baylor versus Broad. We compare the average sample depth for all non-synonymous variants in the two data sets.
(TIF)

Table S1 Comparison of seven individuals called by both Baylor and Broad under different filters.
(PDF)

Table S2Genomic control 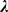 and 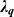 based on different types of PC adjustment.(PDF)

Table S3Genes with p-value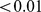 from the SKAT or Burden Test.(XLSX)

Table S4 The p-values of genes which have two or more *de novo* nonsense or missense mutations as reported in [5].
(XLSX)

Table S5 The p-values of 114 ASD genes.
(XLSX)

Table S6 The required sample sizes by applying meta- and mega-analysis.
(PDF)

Table S7 Classification tree results for heterozygote calls.
(PDF)

Text S1 Additional Information Regarding Methods. Part A gives additional information about sequencing, including data generation and quality control. Part B gives the mathematical exposition of mega- and meta-analysis. Part C provides details for association analysis.
(PDF)
