# Supplementary figures and images for: Analysis of Rare, Exonic Variation amongst Subjects with Autism Spectrum Disorders and Population Controls
Source: PLoS Genet. 2013 Apr 11;9(4):e1003443. doi: 10.1371/journal.pgen.1003443 (PMC3623759; doi:10.1371/journal.pgen.1003443)

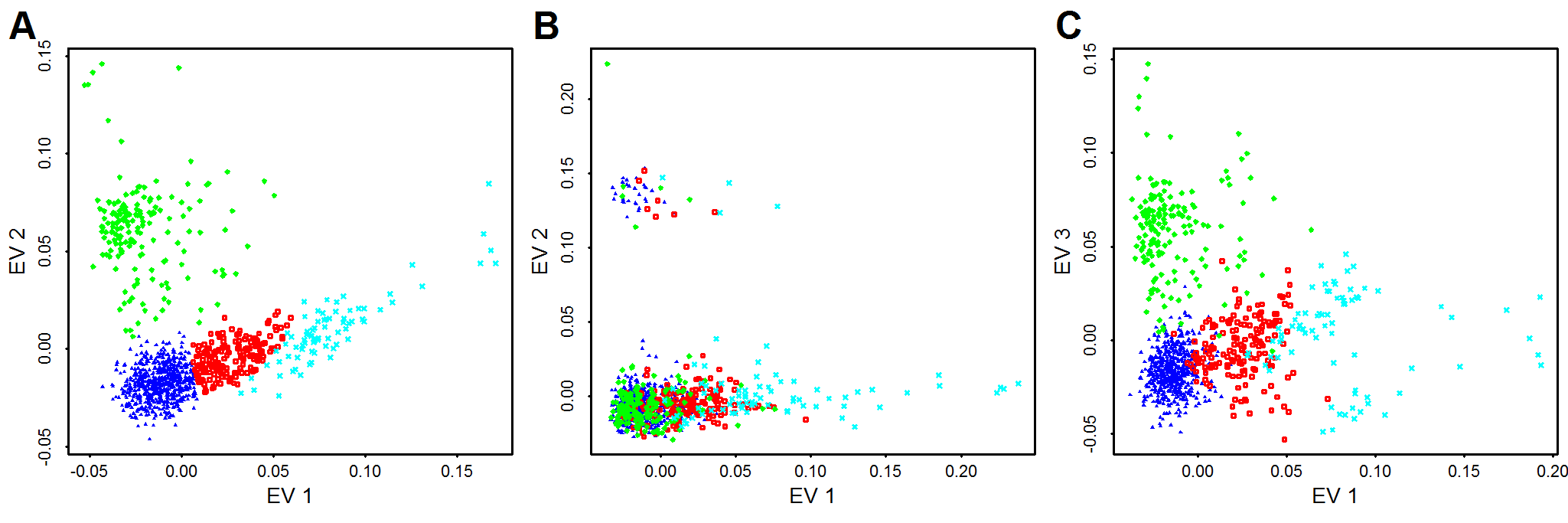

Supplement: Figure S1 — PCA from common variants, low frequency variants and both type of variants for Baylor samples. Eigen-vectors are obtained by applying PCA to all common variants that have no missingness (14,702 variants) (A), all low frequency variants that have no missingness (8783 variants) (B), and both type of variants (C). The colors are obtained by clustering individuals based on their coordinates in panel (A) using model based clustering [51]. (A) and (B) are the first eigen-vector versus second eigen-vector for Baylor samples. (C) is the first eigen-vector versus second eigen-vector for Baylor samples. (TIF) [file pgen.1003443.s001.tif]

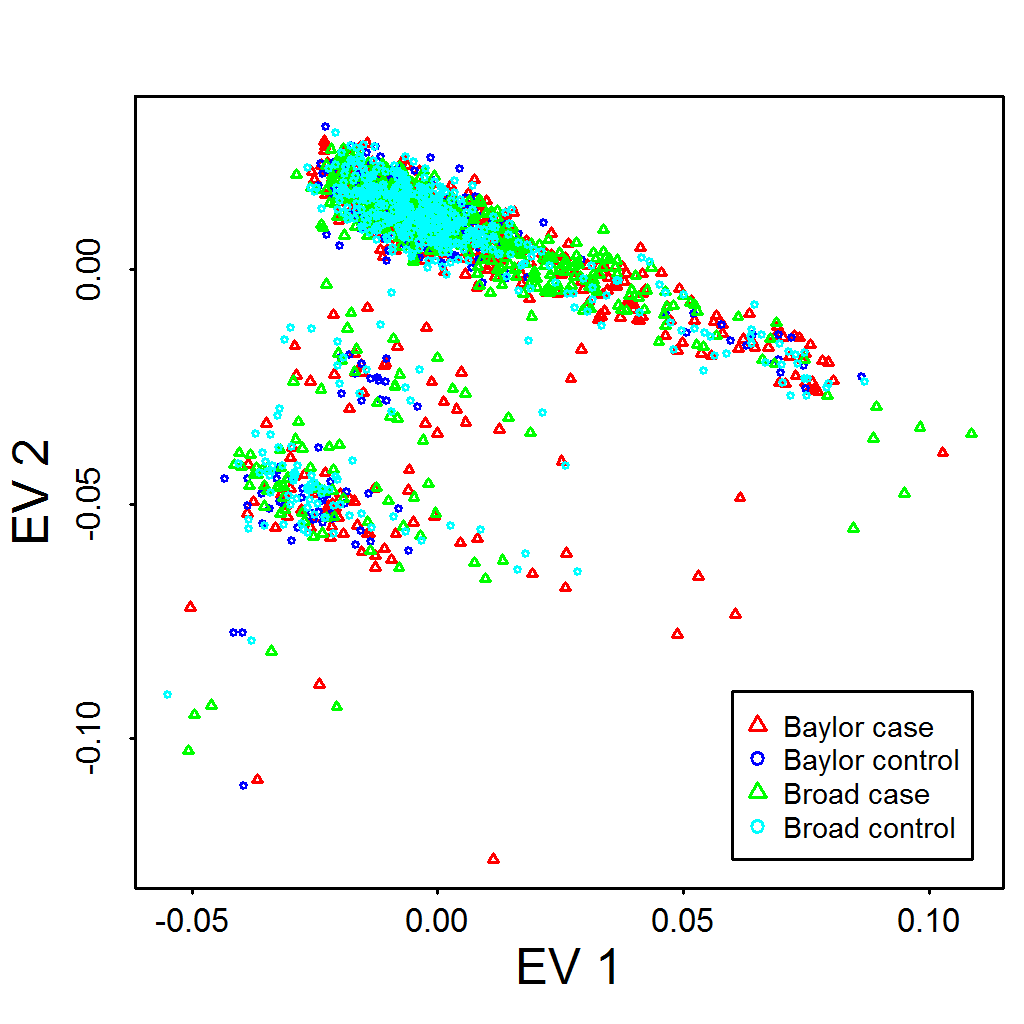

Supplement: Figure S2 — PCA of Baylor and Broad samples together. first eigen-vector versus second eigen-vector for Broad and Baylor samples. (TIF) [file pgen.1003443.s002.tif]

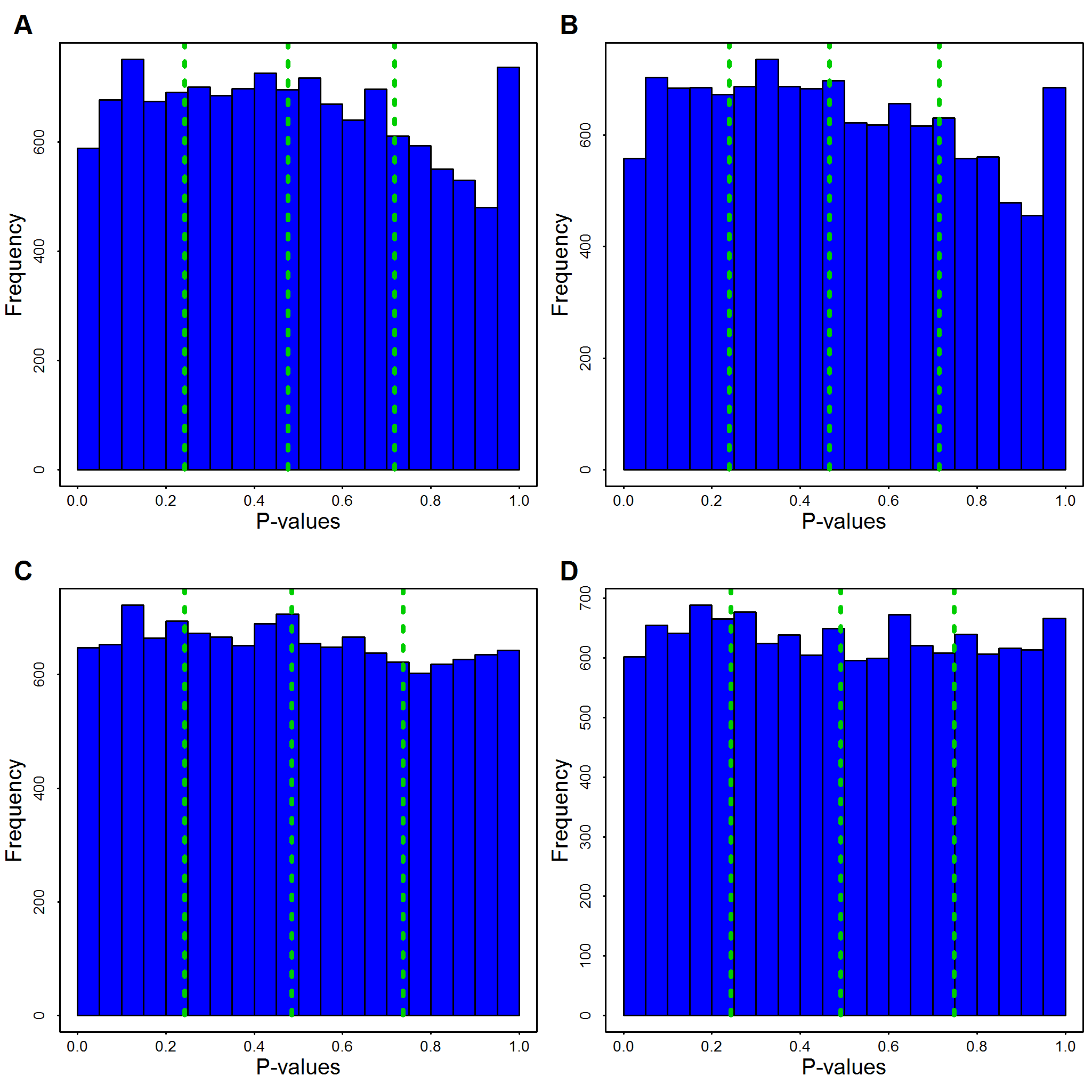

Supplement: Figure S3 — Histogram of p-values for SKAT and Burden Test. (A) and (B) are SKAT p-values for Broad and Baylor samples, respectively. (C) and (D) are Burden test p-values for Broad and Baylor samples, respectively. Green vertical lines are the 25%, 50% and 75% quantiles of p-values. (TIF) [file pgen.1003443.s003.tif]

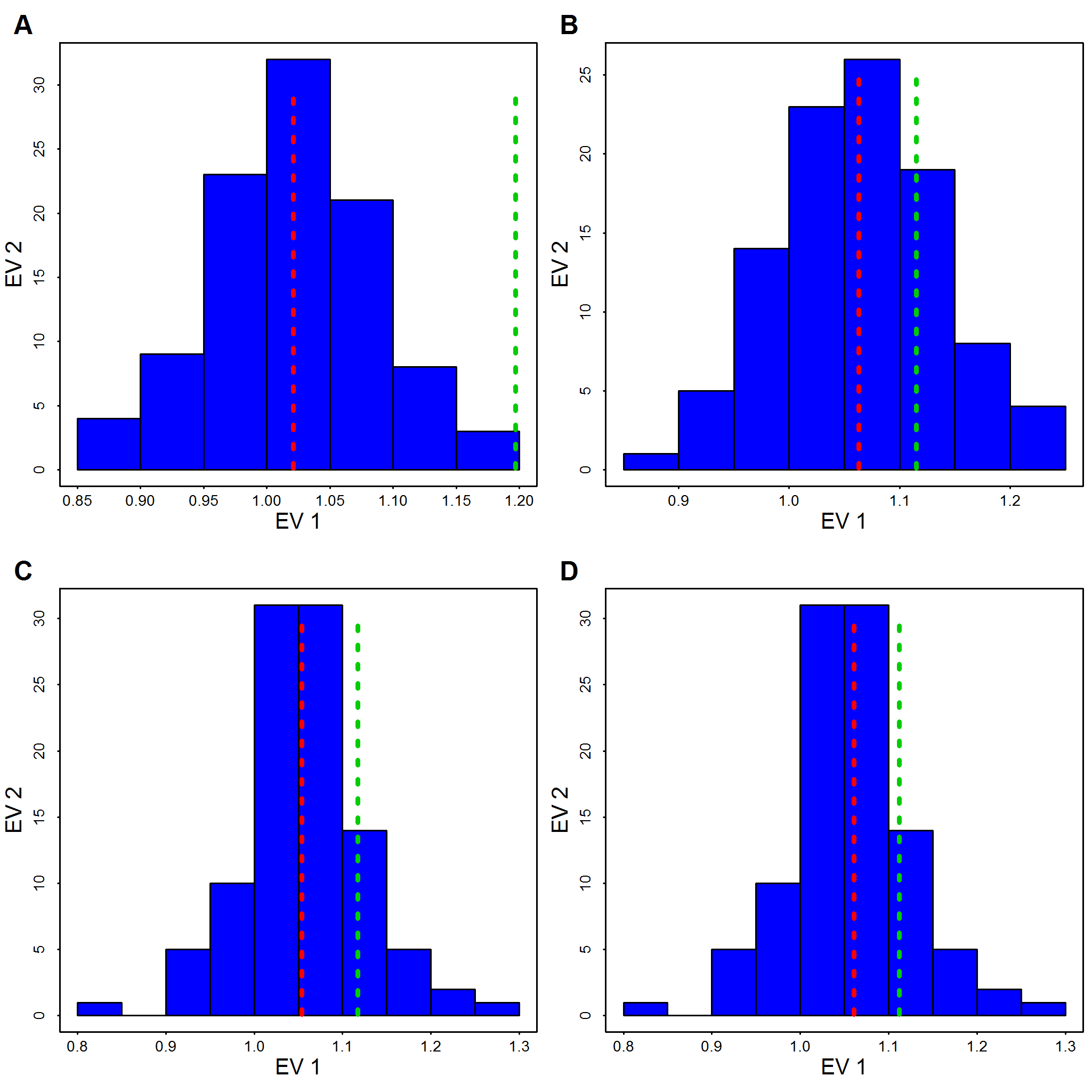

Supplement: Figure S4 — Distribution of the genomic control factor . By permuting case/control status 100 times the distribution of is obtained based on the 1000 largest genes. The red line shows the mean of the permutation distribution and the green line shows obtained from the data using (A) Broad SKAT p-values obtained without eigenvectors; (B) Broad SKAT p-values, with CVs eigenvectors, (C) Broad SKAT p-values, with LFVs eigenvectors; and (D) Broad SKAT p-values, with CVs plus LFVs eigenvectors. (TIF) [file pgen.1003443.s004.tif]

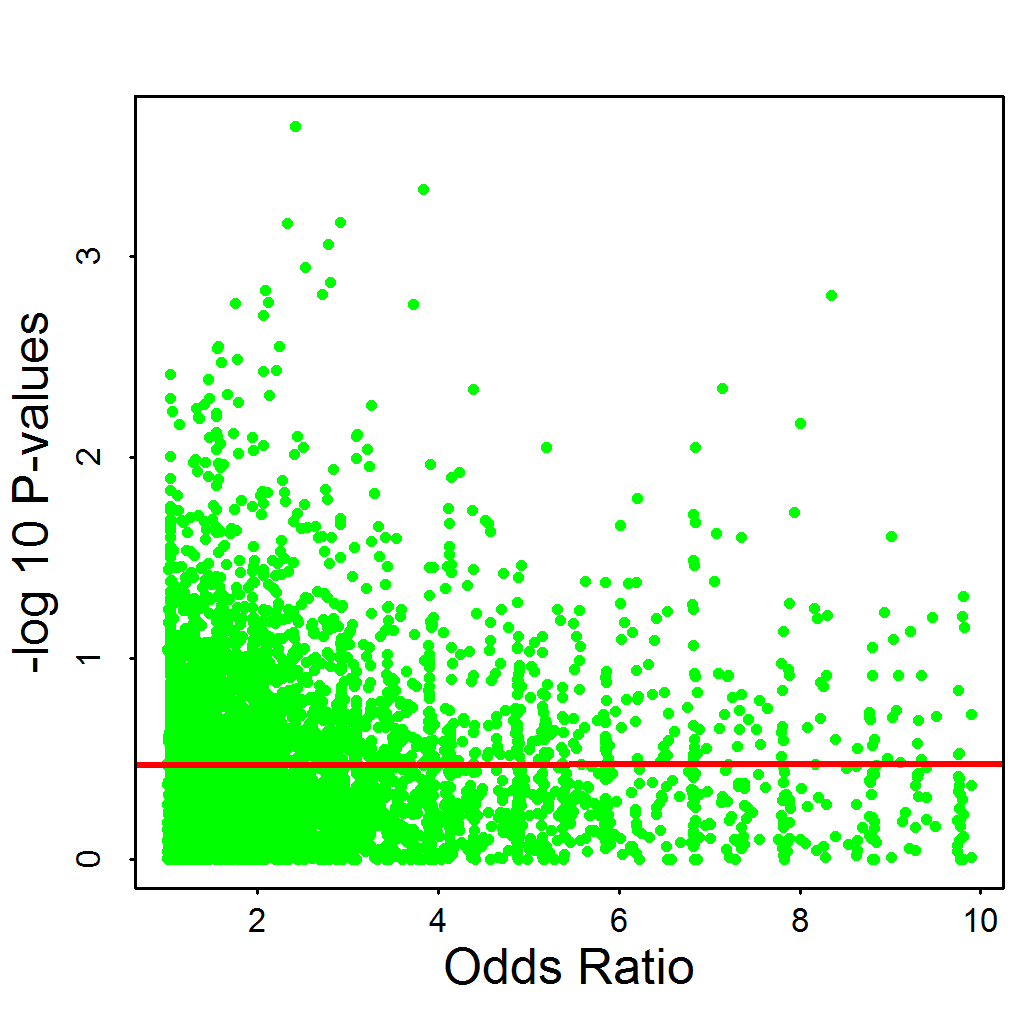

Supplement: Figure S5 — P-values versus Missingness. We used 5500 genes to make this plot. For each gene, we calculate the -log 10 p-values and the odds ratio of missingness in case and control. The red line is the fitted line of these 5500 observations. (TIF) [file pgen.1003443.s005.tif]

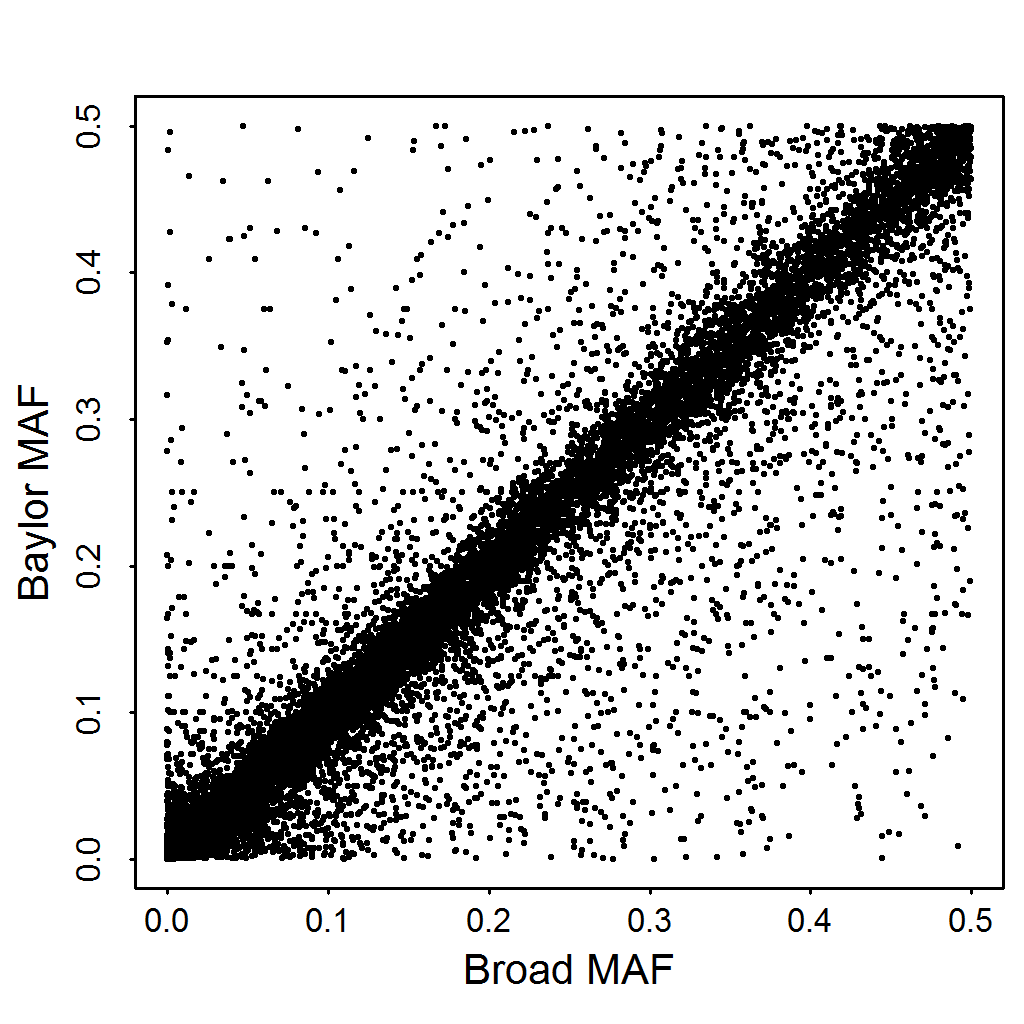

Supplement: Figure S6 — MAF Comparison: Baylor versus Broad. We compare the MAF for 72,758 shared non-synonymous variants in the two data sets. (TIF) [file pgen.1003443.s006.tif]

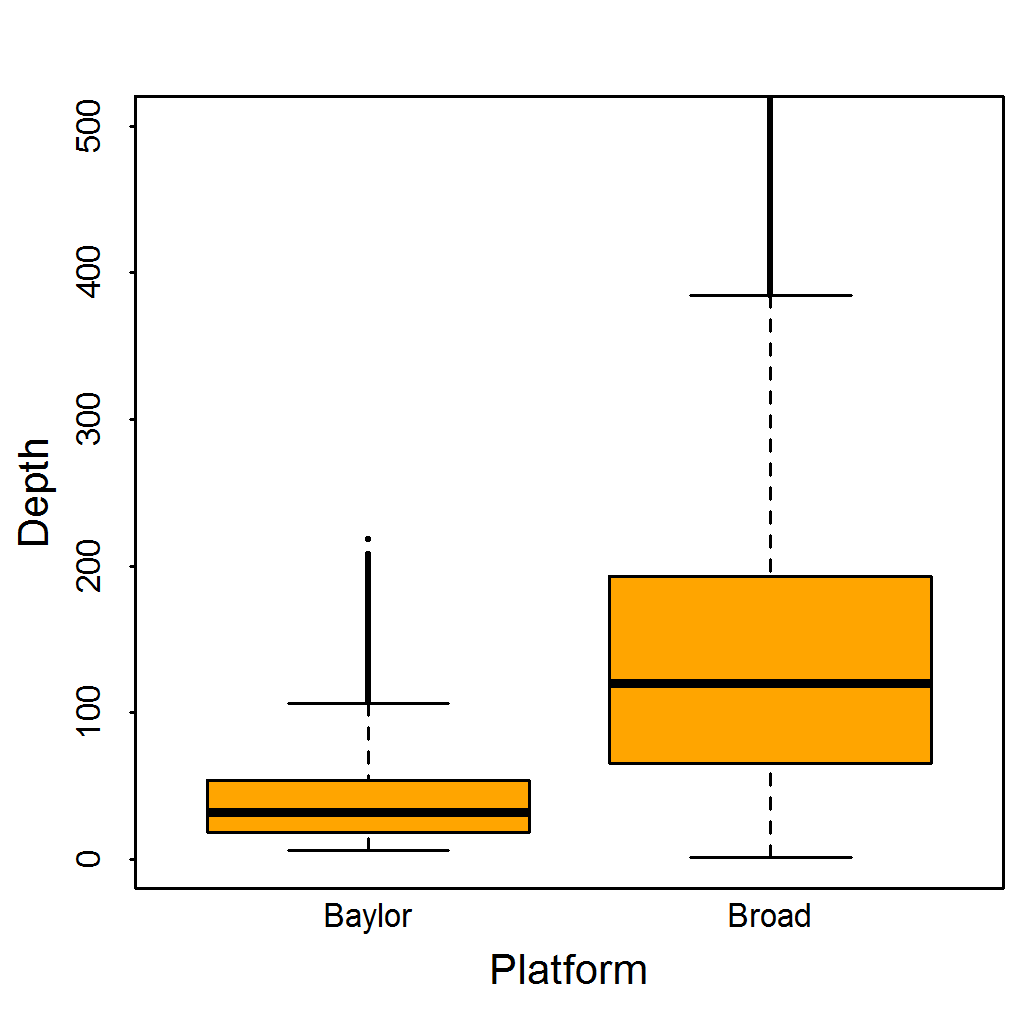

Supplement: Figure S7 — Depth Comparison: Baylor versus Broad. We compare the average sample depth for all non-synonymous variants in the two data sets. (TIF) [file pgen.1003443.s007.tif]
