# Supplementary material for: Analysis of Rare, Exonic Variation amongst Subjects with Autism Spectrum Disorders and Population Controls
Source: PLoS Genet. 2013 Apr 11;9(4):e1003443. doi: 10.1371/journal.pgen.1003443 (PMC3623759; doi:10.1371/journal.pgen.1003443)
Supplement: Table S1 — Comparison of seven individuals called by both Baylor and Broad under different filters. (PDF) [file pgen.1003443.s008.pdf]

Table S1. Comparison of seven individuals called by both Baylor and Broad under different filters.

	Filter PASS		Filter MISS		Filter DpBal	
Br/Ba	hom	het	hom	het	hom	het
hom	335574	37	296568	12 (32%)	288877	9 (24%)
het	96	1771	49 (51%)	1554 (88%)	42 (43%)	1498 (85%)

	Filter B		Filter C		Filter D	
Br/Ba	hom	het	hom	het	hom	het
hom	278949	6 (16%)	269249	4 (10%)	238325	5 (13%)
het	33 (34%)	1439 (81%)	32 (33%)	1356 (77%)	26 (27%)	1151 (65%)

Note: Using all the non-synonymous rare variants called both by Baylor and Broad. Filter PASS includes all variants that score a "Pass" based on GATK, Filter MISS: missingness $< 10\%$, Filter DpBal: missingness $< 10\%$, $\eta > 10$ & $\xi < 0.85$ for Baylor, $\eta > 10$ & $\xi < 0.75$ for Broad; Filter B: Missingness $< 10\%$, $\eta > 10$ & $\xi < 0.75$; Filter C: Missingness $< 10\%$, $\eta > 12$ & $\xi < 0.75$ for Baylor, $\eta > 17$ & $\xi < 0.66$ for Broad; Filter D: Missingness $< 10\%$, $\eta > 10$ & $\xi < 0.66$.
