# Supplementary material for: Analysis of Rare, Exonic Variation amongst Subjects with Autism Spectrum Disorders and Population Controls
Source: PLoS Genet. 2013 Apr 11;9(4):e1003443. doi: 10.1371/journal.pgen.1003443 (PMC3623759; doi:10.1371/journal.pgen.1003443)
Supplement: Table S2 — Genomic control and based on different types of PC adjustment. (PDF) [file pgen.1003443.s009.pdf]

Table S2: Genomic control λ and λ_q based on different types of PC adjustment

	SKAT				Burden			
	no PCA	CVs	LFVs	CV+LFV	no PCA	CVs	LFVs	CV+LFV
Broad λ	1.197	1.115	1.117	1.112	1.109	1.070	1.077	1.088
Broad λ_q	1.064	1.032	1.020	1.028	1.059	1.031	1.039	1.031
Baylor λ	1.251	1.163	1.151	1.148	1.146	1.037	1.029	1.036
Baylor λ_q	1.107	1.046	1.056	1.043	1.094	1.027	1.010	1.023
Mega λ	1.298	1.188	1.166	1.187	1.195	1.107	1.112	1.112
Mega λ_q	1.176	1.078	1.081	1.072	1.151	1.047	1.068	1.069
Meta λ	1.322	1.200	1.205	1.201	1.175	1.082	1.103	1.098
Meta λ_q	1.145	1.089	1.101	1.098	1.104	1.036	1.034	1.050

Note: These analyses are restricted to the genes that have more than 4 minor alleles in the samples used in each study. λ and λ_q are calculated based on the median and the 1st quantile of the p-value distribution, respectively. CVs PC adjustment is based on the common variants eigen-vectors. LFVs PC adjustment is based on the low frequency variants eigen-vectors. CV+LFV PC adjustment is based on the common variants plus low frequency variants eigen-vectors.
