# Supplementary material for: Analysis of Rare, Exonic Variation amongst Subjects with Autism Spectrum Disorders and Population Controls
Source: PLoS Genet. 2013 Apr 11;9(4):e1003443. doi: 10.1371/journal.pgen.1003443 (PMC3623759; doi:10.1371/journal.pgen.1003443)
Supplement: Table S6 — The required sample sizes by applying meta- and mega-analysis. (PDF) [file pgen.1003443.s013.pdf]

Table S6: The required sample sizes to have good power by applying meta- and mega-analysis

	Power> 0.5		Power> 0.8	
	Meta	Mega	Meta	Mega
Scenario A	3050	2360	3850	3060
Scenario B	4030	3130	5090	4000
Scenario C	4450	3370	5550	4260

Note: Required sample sizes are calculated based on the theoretical power function of meta- and mega- analysis in Equation S3 and Equation S4. The p-value threshold is 10^{-5}). Scenario A: the gene has 15 variants, each with MAF = .0025, for which all have odds ratio of 4. Scenario B: the gene has 20 variants, each with MAF = 0.005, for which 10 have odds ratio of 3; Scenario C: the gene has 40 variants, each with MAF = .005, for which 30 have odds ratio of 2.
