# Supplementary material for: Analysis of Rare, Exonic Variation amongst Subjects with Autism Spectrum Disorders and Population Controls
Source: PLoS Genet. 2013 Apr 11;9(4):e1003443. doi: 10.1371/journal.pgen.1003443 (PMC3623759; doi:10.1371/journal.pgen.1003443)
Supplement: Table S7 — Classification tree results for heterozygote calls. (PDF) [file pgen.1003443.s014.pdf]

**Table S7. Classification tree results for heterozygote calls.**

| | Stringent Filter | | Lenient Filter | |
|---|---|---|---|---|
| Predicted/Confirmed | Yes | No | Yes | No |
| Yes | 83 | 45 | 87 | 74 |
| No | 4 | 390 | 0 | 361 |

Note: Stringent Filter: missingness $< 10\%$, $\eta > 17$ & $\xi < 0.66$; Lenient Filter: missingness $< 10\%$, $\eta > 10$ & $\xi < 0.75$.
