# Supplementary material for: Analysis of Rare, Exonic Variation amongst Subjects with Autism Spectrum Disorders and Population Controls
Source: PLoS Genet. 2013 Apr 11;9(4):e1003443. doi: 10.1371/journal.pgen.1003443 (PMC3623759; doi:10.1371/journal.pgen.1003443)
Supplement: Text S1 — Additional Information Regarding Methods. Part A gives additional information about sequencing, including data generation and quality control. Part B gives the mathematical exposition of mega- and meta-analysis. Part C provides details for association analysis. (PDF) [file pgen.1003443.s015.pdf]

Text S1: Additional Information Regarding Methods

A. Data Generation and Quality Control

The institutional review board of all participating institutions approved this study and written informed consent or assent, as appropriate, was obtained from all subjects.

Exome capture and sequencing at Broad was performed as follows. Genomic DNA was sheared to 200-300 bp using a Covaris Acoustic Adaptor. Fragments were end-repaired, dA-tailed, and sequencing adaptor oligonucleotides ligated using reagents from New England BioLabs. Libraries were barcoded using the Illumina index read strategy, which uses six-base sequences within the adapter that are sequenced separately from the genomic DNA insert. The DNA library was subsequently enriched for sequences with 5' and 3' adapters by PCR amplification using with primers complementary to the adapter sequences (ligation-mediated PCR, LM-PCR). Exons were captured using the Agilent 38Mb SureSelect v2. In some cases, barcoded libraries from 2-4 subjects were mixed prior to hybridization with the capture reagent. After capture, another round of LM-PCR was performed to generate enough DNA to sequence. Libraries were sequenced using an IlluminaHiSeq2000.

At Baylor, genomic DNA was sheared into fragments of approximately 120 base pairs with the Covaris S2 or E210 system. Fragments were processed through DNA End-Repair and A-tailing, and the resulting fragments were ligated with BCM-HGSC-designed Truncated-TA (TrTA) P1 and TA-P2 adapters with the NEB Quick Ligation Kit. Solid Phase Reversible Immobilization bead cleanup was used to purify the adapted fragments, after which nick translation and Ligation-Mediated PCR was performed using Platinum PCR Supermix HIFi. The pre-capture libraries were hybridized to NimbleGen EZ Exome v2, VCRome v1, or VCRome v2.1 probes, either in solution or with solid-phase capture chips, and then amplified. In some cases, barcoded capture libraries were pooled in sets of 4 samples after post-capture amplification. Libraries were sequenced on the Life Technologies SOLiD platform using both 50bp fragment and 50x35bp paired-end run formats.

At Broad, data was processed with Picard (http://picard.sourceforge.net/), which utilizes base quality-score recalibration and local realignment at known indels and BWA for mapping reads to hg19. SNVs were called using GATK [**?**]. The variable sites that we have considered in analysis are restricted to those that pass GATK standard filters to eliminate SNVs with strand-bias, low quality for the depth of sequencing achieved, homopolymer runs, and SNVs near indels. At Baylor, data was processed with

Picard and reads mapped to hg18 using Bfast [?]. The quality score recalibration and indel realignment was performed using GATK, followed by single nucleotide variant (SNV) identification using AtlasSNP 2 software [?].

Prior to filtering we compare the minor allele frequency (MAF) of SNVs for Baylor and Broad data sets (Fig. S6). These two platforms differ in read depth (Fig. S7).

A model for filtering of SNVs was developed to improve the quality of the data and facilitate combining of data from 2 platforms. The misclassification of a common homozygote as a heterozygote can largely reduce the power of rare variants association tests [?]. In our data some heterozygous calls were based on very low and unbalanced depth. Filtering out these low-quality heterozygous calls should improve power.

To create a suitable filter based on the data, we choose the 104 trios from the Broad data set [?]. Prior to filtering we found 2272 potential *de novos* mutations among 104 trios, but this greatly exceeds expectation and most of these are false calls. Because low quality parental genotype calls can lead to false *de novo* discoveries, we first remove those candidates that are likely due to errors at this level. If one parent is heterozygous, but his/her genotype call is common homozygous, then a false positive *de novo* is created in this position. Therefore, we first use a simple filter to delete the *de novo* discoveries likely tracing to low quality homozygous calls in parents, namely that the GQ score must be larger than 30 and the balance must be larger than 95%. By this filter we reduce the number of potential *de novos* from 2272 to 522.

Based on a parsimonious filter for complete trios, Neale et al. [?] greatly reduced this list and identified 87 true *de novos*, which were confirmed by Sanger sequencing. We label the remaining 435 heterozygotes to be false calls. Now we use the data from the ASD probands of these trios to develop a filter. Here we used the quality variables available to us (depth, balance and genotype quality (GQ)) to build a prediction model. Based on this information, and by using classification tree methods we can find a good threshold to filter the false minor allele calls. The filter selected by 10-fold cross validation keeps minor allele calls that have depth larger than 17 and balance smaller than 0.66. The misclassification rate is 0.09, but 4 of the 87 validated *de novo* variants were missed with this stringent filter. A slightly less stringent filter with depth larger than 10 and balance smaller than 0.75 also gave good classification results, with no false negatives. Table S2 shows the classification results of the stringent and lenient filter.

We compared the seven common samples processed at both sites using 4 varieties of filters plus 2 very minimal filters (Table S1). First we identified all non-synonymous rare variants. Applying the stringent

filter described previously ($\eta > 17$ and $\xi < .66$) to both data sets we found that Baylor had far too few heterozygous calls and the concordance rate across sites was greatly reduced. This is presumably due to the lower depth of the Baylor reads overall. Clearly we need to use a less stringent cutoff for depth and balance if we wish to obtain comparable call rates; however we do not want to do so if it leads to a high rate of false heterozygous calls in Baylor relative to the filtered Broad calls. We considered 4 filters to select a cutoff that produced a high concordance rate with a low rate of potential false positives in Baylor versus Broad. With a Baylor filter of depth > 10 and balance < .85 we achieved this goal, reducing the mismatch rate to .017% with only 9 heterozygotes called in Baylor, but not in Broad.

B. Mathematical exposition of mega- and meta-analysis

To obtain equation (Eqn. 4), note that

$$Q = \sum_{j=1}^{d} [\mathbf{T}_j'(\mathbf{y} - \hat{\boldsymbol{\mu}})]^2 = \sum_{j=1}^{d} S_j^2,$$

where S_j is the score of the jth variant. The j'th score can be expressed as

$$\begin{aligned} S_j &= \mathbf{T}_j'(\mathbf{y} - \hat{\boldsymbol{\mu}}) \\ &= (T_{1j}, ..., T_{nj})'(\sum_k \beta_k G_{1k}, ..., \sum_k \beta_k G_{nk}) + (T_{1j}, ..., T_{nj})'(\epsilon_1, ..., \epsilon_n) \\ &= \sum_i^n \sum_k \beta_k T_{ij} G_{ik} + \sum_1^n T_{ij}\epsilon_i \\ &= \sum_i^n \sum_k \beta_k T_{ij}(T_{ik}\sqrt{2p_k(1-p_k)n} + 2p_k) + \sum_1^n T_{ij}\epsilon_i. \end{aligned}$$

hence $S_j \sim N(\sum_k \sqrt{2p_k(1-p_k)n} I\{j = k\}\beta_k, 1)$.

Under the null hypothesis, $\beta_j = 0$ for all j, therefore $Q \sim \chi_d^2$, and under the alternative hypothesis,

$$Q \sim \chi_d^2(\delta), \quad \delta = \sum_k 2p_k(1-p_k)n\beta_k^2.$$

To obtain equation (Eqn. 5), note that the score test statistics can be written as

$$Q = \tilde{\mathbf{Y}}^T K \tilde{\mathbf{Y}} = \sum_{j=1}^{d} S_j^2,$$

where $\tilde{Y}_i = (Y_i - \hat{\mu})/\sqrt{(\hat{\mu})(1-\hat{\mu})}$, $K = T^T T$ and by assumption $E[K] = \mathbf{I}$. Now consider

$$S_j = \sum_{i=1}^{n} T_{ij}(Y_i - \hat{\mu})/\sqrt{(\hat{\mu}(1-\hat{\mu})}.$$

Under the null hypothesis,

$$Y_i \sim \text{Bernoulli}(\hat{\mu})$$

By the Lindeberg central limit theorem (CLT), if T_j is not dominated by a few elements, then $\sum_i T_{ij}\tilde{Y}_i$ is approximately independent standard normal distribution. Therefore

$$Q \sim \chi^2_d$$

Under the alternative hypothesis,

$$Y_i \sim \text{Bernoulli}(\tilde{\mu}),$$

where $\tilde{\mu} = \text{logit}^{-1}(\alpha + \sum_k \beta_k G_{ik})$. By Taylor's expansion, we have

$$\text{logit}^{-1}(x) = \frac{\exp(x)}{1+\exp(x)} \approx \frac{1}{2} + \frac{1}{4}x,$$

so

$$\begin{aligned}
\tilde{\mu}_i - \hat{\mu} &= \text{logit}^{-1}(\alpha + \sum_k \beta_k G_{ik}) - \text{logit}^{-1}(\alpha) \\
&\approx \frac{1}{2} + \frac{1}{4}(\alpha + \sum_k \beta_k G_{ik}) - \frac{1}{2} - \frac{1}{4}\alpha \\
&= \frac{1}{4}\sum_k \beta_k G_{ik}.
\end{aligned}$$

By Lindeberg CLT the distribution of S_j is:

$$S_j \sim N(\sum_k \frac{1}{4} I\{j = k\}\sqrt{2p_k(1-p_k)n}\beta_k, 1),$$

and therefore $Q \sim \chi^2_d(\delta)$, where $\delta = \frac{1}{8}\sum_k p_k(1-p_k)n\beta_k^2$.

Next we seek an expression of power of meta- and mega-analysis. For the meta-analysis, we first calculate the p-value of Q_1 and Q_2 under the null distribution.

$$Pr_1 = 1 - F(Q_1), Pr_2 = 1 - F(Q_2),$$

where F is the CDF of χ^2_p distribution. Since

$$\frac{\Phi^{-1}(Pr_1) + \Phi^{-1}(Pr_2)}{\sqrt{2}} \sim N(0, 1) \tag{1}$$

under the null hypothesis; it can be used as the test statistics of meta-analysis. Since the pdf of χ^2_d is complicated, we use a normal approximation, due to R.A. Fisher: $\sqrt{2\chi^2_d}$ is approximately equivalent to

$N(\sqrt{2d-1}, 1)$. Therefore Pr_1 and Pr_2 can be rewritten as

$$Pr_1 = 1 - \Phi(\sqrt{2Q_1} - \sqrt{2d-1}), Pr_2 = 1 - \Phi(\sqrt{2Q_2} - \sqrt{2d-1}).$$

So now we have two approximate statistics for meta- and mega-analysis

$$\text{Meta:} \quad Q'_t = \sqrt{Q_1} + \sqrt{Q_2} - \sqrt{4d-2},$$
$$\text{Mega:} \quad Q'_g = \sqrt{2Q_g} - \sqrt{2d-1},$$

Under α level type I error, the power of meta- and mega-analysis are as followed:

$$\text{Power}_t = \int_{q_{1-\alpha}}^{\infty} f_{Q'_t}(x)dx,$$
$$\text{Power}_g = \int_{q_{1-\alpha}}^{\infty} f_{Q'_g}(x)dx,$$

where $q_{1-\alpha}$ is the $1-\alpha$ quantile of normal distribution, and $f_{Q'_t}$, $f_{Q'_g}$ are the density of Q'_t and Q'_g.

Because the pdf of a non-central chi-distribution is very complicated, we apply another approximation due to [?]:

$$P(\chi_d^2(\delta) < x) = \Phi\left\{\frac{(\frac{x}{d+\delta})^{\frac{1}{3}} - 1 + \frac{2}{9}\cdot\frac{1+b}{d+\delta}}{\sqrt{\frac{2}{9}\cdot\frac{1+b}{d+\delta}}}\right\}, \tag{2}$$

where $b = \delta/(d+\delta)$.

Let $c = \delta/d$ and plug

$$x_t = (q_\alpha + \sqrt{4d-2} - \sqrt{q_2})^2$$
$$x_g = \frac{1}{2}(q_\alpha + \sqrt{2d-1})^2$$

into (Eqn. S2), where

$$q_2 = \left(\sqrt{\frac{2}{9}\frac{2c+1}{(c+1)^2 d}}x + 1 - \frac{2}{9}\frac{2c+1}{(c+1)^2 d}\right)^3 (c+1)d$$

and x follows standard normal distribution. We obtain the power of meta- and mega-analysis under the level of α:

$$Power_t = 1 - \int \Phi\left\{\frac{(\frac{x_t}{(c+1)d})^{\frac{1}{3}} - 1 + \frac{2}{9}\cdot\frac{2c+1}{(c+1)^2 d}}{\sqrt{\frac{2}{9}\cdot\frac{2c+1}{(c+1)^2 d}}}\right\}\phi(x)dx, \tag{3}$$

$$Power_g = 1 - \Phi\left\{\frac{(\frac{\frac{1}{2}(q_\alpha+\sqrt{2d-1})^2}{(2c+1)d})^{\frac{1}{3}} - 1 + \frac{2}{9}\cdot\frac{4c+1}{(2c+1)^2 d}}{\sqrt{\frac{2}{9}\cdot\frac{4c+1}{(2c+1)^2 d}}}\right\}. \tag{4}$$

Some insight can be gained by noting that Q'_t is well approximated by $\sqrt{2(Q_1+Q_2)} - \sqrt{4d-1}$.

C. Association Analysis.

When merging data files from separate sources a substantial fraction of the observations can be missing due to the filtering process. However, due to the nature of the test statistic, missing values have a negligible effect on inference. This can be best explained in the context of the C-alpha test, which is equivalent to the SKAT test under certain conditions. Recall that if a variant is observed z times in cases and $m - z$ times in controls, the C-alpha statistic compares the distribution of $(z, m - z)$ to a binomial distribution with probability of success equal to the fraction of cases in the sample. The test then accumulates this information across variants in the gene. Missing data would have no effect on the test statistic provided missingness was equally distributed over cases and controls. In our analysis missingness varied by site (Baylor and Broad), and by case/control status (Table 3). In practice, SKAT imputes missing values using single imputation. Because our analysis is restricted to variants with MAF $< .01$, this is essentially equivalent to imputing missing values with the reference allele. We compared results obtained with and without imputation and found this procedure had negligible effect on the outcome.

Due to inaccuracies in the P-values for genes with very few variants and numerous covariates, we include only the P-values for the genes that have minor alleles counts (MACs) greater than 4. With this filter, we have 12,676 genes in Baylor and 13,119 genes in Broad. For those genes clearly not associated with the phenotype (p-values $> .5$) we found that SKAT tended to report p-values biased downward toward .5, causing an apparent, but uninteresting, inflation in the genomic control (GC) inflation factor. Hence we calculate the GC factor using the third quantile of $\{F^{-1}(1 - \text{p-value})\}$, where F^{-1} is the inverse CDF of χ^2_1 distribution, rather than the median. Dividing this quantity by its expectation, which is equal to 1.32, leads to a more robust, but equivalent measure of the GC factor.

For AASC data analysis, weights are set at $\sqrt{\omega_j} = \text{Beta}(p_j, 1, 25)$. We use SKAT to calculate the p-values for Baylor, Broad and the merged data sets. We combine all singleton variants as a super-variant. For meta-analysis the weighted Z-score method is applied to calculate the combination of two p-values from Broad and Baylor, and we only report the p-values for the genes with MACs greater than zero in both Broad and Baylor (15,640 genes).

Although SKAT overcomes the drawbacks of the burden tests, as discussed in [**?**], it is not as powerful as the burden tests if all variants have the same association direction. For this reason we also used the

burden test. We did not try SKAT-O [?] which automatically finds the most powerful test in a family of tests ranging from pure-burden to the original SKAT test. Given that we applied both tests to the full set of genes, but did not find any signals in our data, we do not expect to realize any additional benefit of SKAT-O in this setting.

Supplemental Acknowledgement

The collection of data and biomaterials in one project that participated in the National Institute of Mental Health (NIMH) Autism Genetics Initiative has been supported by National Institute of Health grants MH52708, MH39437, MH00219, and MH00980; National Health Medical Research Council grant 0034328; and by grants from the Scottish Rite, the Spunk Fund, Inc., the Rebecca and Solomon Baker Fund, the APEX Foundation, the National Alliance for Research in Schizophrenia and Affective Disorders (NARSAD), the endowment fund of the Nancy Pritzker Laboratory (Stanford); and by gifts from the Autism Society of America, the Janet M. Grace Pervasive Developmental Disorders Fund, and families and friends of individuals with autism. The Principal Investigators and Co-Investigators were: Stanford University, Stanford: Neil Risch, Ph.D., Richard M. Myers, Ph.D., Donna Spiker, Ph.D., Linda J. Lotspeich, M.D., Joachim Hallmayer, M.D., Helena C. Kraemer, Ph.D., Roland D. Ciaranello, M.D., Luca L. Cavalli-Sforza, M.D., University of Utah, Salt Lake City: William M. McMahon, M.D. and P. Brent Petersen. The Stanford team is indebted to the parent groups and clinician colleagues who referred families. The Stanford team extends our gratitude to the families with individuals with autism who were our partners in this research.

The collection data and biomaterials come from the Autism Genetic Resource Exchange (AGRE) collection. This program has been supported by a National Institute of Health (grants MH64547 and 1U24MH081810) and Autism Speaks, Inc. (formerly the Cure Autism Now Foundation). The Principal Investigator of grant MH64547 is Daniel H. Geschwind, M.D., Ph.D. (UCLA). The Co-Principal Investigators include Stanley F. Nelson, M.D., and Rita Cantor, Ph.D. (UCLA), Christa Lese Martin, Ph.D. (U. Chicago), T. Conrad Gilliam, Ph.D. (Columbia). Co- investigators include Maricela Alarcon, Ph.D., Kenneth Lange, Ph.D., Sarah J. Spence M.D., Ph.D. (UCLA), David H. Ledbetter Ph.D. (Emory) and Hank Juo, M.D., Ph.D. (Columbia).

The Principal Investigator of grant 1U24MH081810 is Clara M. Lajonchere, Ph.D. (USC). The Co-Principal Investigators include Steven Moldin, Ph.D. (USC), Janet Miller, J.D., Ph.D. (Autism Speaks),

Mark Urata, M.D. (CHLA), Constantinos Sioutas, Ph.D. (USC), David Amaral, Ph.D. (UC Davis), Curtis Deutsch, Ph.D. (UMASS).

Scientific oversight of the AGRE program is provided by the AGRE steering committee: Dan Geschwind, M.D., Ph.D., UCLA; Maja Bucan, Ph.D., University of Pennsylvania; W. Ted Brown, M.D., Ph.D., F.A.C.M.G., N.Y.S. Institute for Basic Research in Developmental Disabilities; Rita M. Cantor, Ph.D., UCLA; John N. Constantino, M.D., Washington University School of Medicine, St. Louis; T. Conrad Gilliam, Ph.D., University of Chicago; Martha Herbert, M.D., Ph.D., Harvard Medical School; Clara Lajonchere, Ph.D., Autism Speaks; David H. Ledbetter, Ph.D., Emory University; Christa Lese-Martin, Ph.D., Emory University; Janet Miller, J.D., Ph.D., Autism Speaks; Stanley F. Nelson, M.D., UCLA; Gerard D. Schellenberg, Ph.D., University of Pennsylvania; Carol A. Samanago-Sprouse, Ed.D., George Washington University; Sarah Spence, M.D., Ph.D., NIMH; Matthew State, M.D., Ph.D., Yale University; Rudolph E. Tanzi, Ph.D., Massachusetts General Hospital.

The collection of data and biomaterials in another project has been supported by National Institutes of Health grant MH55135 (Collaborative Linkage Study of Autism). The Principal Investigator was Susan E. Folstein, M.D. (Tufts University/New England Medical Center, Boston, MA), and her key Clinical and Phenotypic Coordinators were Brian Winklosky and Beth Rosen-Sheidley, M.S., C.G.C. Co-Investigators included James S. Sutcliffe, Ph.D. and Jonathan L. Haines, Ph.D. (Vanderbilt University, Nashville, TN).

The collection of data and biomaterials in another project has been supported by National Institute of Health grant MH55284. The Principal Investigator and Co-Investigators were: University of North Carolina, Chapel Hill: Joseph Piven, M.D., University of Iowa, Iowa City: Val Sheffield, M.D., Ph.D., Veronica Vieland, Ph.D. and Thomas Wassink, M.D.

The Shanghai-New Jersey Consortium is currently conducting a privately funded Autism Candidate Gene Study involving researchers at Rutgers University (Department of Genetics) and University of Dentistry and Medicine of New Jersey (UMDNJ) (Center for Advanced Biotechnology and Medicine), New Jersey and The Chinese National Human Genome Center at Shanghai, China. The principal Investigator involved is Jay A. Tischfield, Ph.D. and the Co- Principal Investigators in New Jersey include Lei Yu, Ph.D., Linda M. Brzustowicz, M.D., Neda Gharani, Ph.D., James H. Millonig, Ph.D., Tara Matise, Ph.D., Derek Gordon, Ph.D. and in Shanghai Wei Huang, Ph.D., Ying Wang, Ph.D.

NIDCD funded a program project grant (PO1/U19 DC 03610) that was conducted initially at the Eunice Kennedy Shriver Center in Waltham, MA, and then transferred to Boston University School of

Medicine (Department of Anatomy and Neurobiology). This program project was part of the NICHD/NIDCD funded Collaborative Program of Excellence in Autism (CPEA). The Principal Investigator was Helen Tager-Flusberg, Ph.D. with Susan Folstein as the co-PI. Clinical data were collected by Robert Joseph, Ph.D., Susan Bacalman, M.S.W. and a team of students and research assistants. As part of this program project, a supplement was awarded by NIDCD to collect blood samples from the children enrolled in the program project and their first degree relatives. The collection of the blood samples was coordinated by Nancy Shaffer, B.A.

The University of Washington Autism Center research was funded by a grant from the National Institute of Child Health and Human Development (U19HD34565; Geraldine Dawson, Director), which is part of the NICHD Collaborative Program of Excellence in Autism. The CPEA program project is directed by Geraldine Dawson, Ph.D. (Department of Psychology), with Gerard D. Schellenberg, Ph.D. (Departments of Neurology, and Gerontology and Geriatric Medicine) as molecular biologist, and Ellen M. Wijsman, Ph.D. (Department of Biostatistics and Division of Medical Genetics) as statistical geneticist. The Director of the Data Management and Statistical Core is Robert Abbott, Ph.D. (Department of Educational Psychology) with Jeffery Munson, Ph.D. (UW Autism Center). Associate Director in charge of recruitment and diagnostic data collection is Annette M. Estes, Ph.D. (Department of Psychiatry and Behavioral Sciences, Division of Child Psychiatry).

As part of an NIH supplementary project, Mount Sinai School of Medicine was funded from 2004-2005 to contact participants that previously participated in a family/genetic study of autism. Multiplex and simplex families were included. All participants were reconsented specifically to have their biomaterial and diagnostic assessment data contributed to NIMH repository. Any outstanding diagnostic and cognitive assessments were also collected. Blood samples were collected from affected and unaffected family members. For all affected family members, diagnostic assessments included the ADI-R, ADOS-G, Vineland Adaptive Behavior Scale, the PPVT-III and/or the Leiter International Performance Scale. The Principal Investigator was Dr. Alison McInnes, and Co-Investigators were Drs. Jeremy Silverman and Christopher J. Smith, who also supervised the data collection. The diagnostic data collection was performed by staff members at the Family Studies Research Center at MSSM who were trained and reliable raters on the ADI-R and ADOS-G.

Data and biomaterials collected for the Indiana University Genetic Studies of Autism has been supported by National Institutes of Mental Health grant U10-MH66766-02S1. The Principal Investigator

was Christopher J. McDougle, M.D. (Indiana University) and the Co-Principal Investigator was John I. Nurnberger, Jr., M.D., Ph.D. (Indiana University). Co-Investigators included David Posey, M.D. (Indiana University), Carrie Smiley, R.N., as project coordinator, Sandi Barton and Kurt Williman as research interviewers. We acknowledge assistance form Naomi Swiezy, Ph.D., Kelly Ernsperger, LCSW, and Jennifer Wilerson, R.N.

Data was also provided by Dr. Patricia Rodier and Dr. Christopher Stodgell at the University of Rochester.

The collection of data and biomaterials for Study 41 was done by investigators in the STAART Network under an NIH Grant. The grants focus was the study of the treatment of affective disturbance in children with autism through experiments addressing three specific aims: 1. To determine if the serotonin reuptake inhibitor, citalopram, is effective in the treatment of behavioral disturbance in children with autism. 2. To determine if physiological or genetic markers, measures of family function, or particular pretreatment symptoms are predictive of sensitivity and response to treatment with citalopram. 3. To better understand the response in clinical trials of children with autism by identifying factors influencing parent and clinician ratings of change and to develop new strategies by which to capture the response to therapeutic interventions.

Blood samples were collected between April 1, 2004 and October 30, 2006 and sent to the Genetic Repository from children and their parents participating in the citalopram trial. The citalopram trial was a multi-site randomized controlled trial (RCT) of the selective serotonin reuptake inhibitor (SSRI), citalopram, for the treatment of 149 children ages 5 to 17 with Autistic Spectrum Disorders (ASD) and moderate to severe levels of repetitive behaviors. This work was funded by National Institutes of Health via the following STAART center contracts: Mount Sinai School of Medicine, New York, New York: U54-MH066673, Eric Hollander, MD, principal investigator (PI) 5/1/04-12/11/08), Joseph Buxbaum, PI (12/12/08-4/30/09); University of North Carolina at Chapel Hill: U54-MH066418, Joseph Piven, MD, PI; University of California at Los Angeles: U54-MH068172, Marian Sigman, PhD, PI; Yale University, New Haven, Connecticut: U54-MH066494, Fred Volkmar, MD, PI. Dartmouth Medical School, Hanover, New Hampshire, and Boston University, Boston, Massachusetts: U54-MH066398, Helen Tager-Flusberg, PhD, PI; and DM-STAT, Inc, Boston: U01-HD045023, Kimberly Dukes, PhD, PI. Representatives from NIH included Ann Wagner, Ph.D.; Deborah Hirtz, M.D.; and Louise Ritz, MBA. The principal investigators included Eric Hollander, M.D.; Linmarie Sikich, M.D.; James T. McCracken, M.D.; Lawrence Scahill,

M.S.N., Ph.D.; Joel D. Bregman, M.D.; Craig L. Donnelly, M.D.; and Bryan H. King, M.D. The Data Coordinating Center was led by Kimberly Dukes, Ph.D.

The RCT, NCT00086645, was registered at clinicaltrials.gov prior to onset and was conducted at the following 6 academic medical centers: Mount Sinai School of Medicine, New York, New York; North ShoreLong Island Jewish Health System, New York; University of North Carolina at Chapel Hill; University of California at Los Angeles; Yale University, New Haven, Connecticut; and Dartmouth Medical School, Hanover, New Hampshire.

In addition to the submission of trio blood samples, the following baseline (prior to treatment administration) was collected: Aberrant Behavior Checklist (ABC), Autism Diagnostic Interview- Revised (ADI-R), Autism Diagnostic Observation Schedule (ADOS), Vocabulary (Comprehensive Test of Phonological Processing (CTOPP) and Peabody Picture Vocabulary Test (PPVT)), IQ (Leiter International Performance Scale-Revised (Leiter-R), Mullen Scales of Early Learning, Wechsler Intelligence Scale for Children (WISC IV), Wechsler Abbreviated Scales of Intelligence (WASI) or Stanford-Binet Intelligence Scales 5th addition) and Vineland Adaptive Behaviors.

We extend our gratitude to the children and families who participated in the repository, and the STAART Psychopharmacology Network investigators for data collection.

Study 35 – From 1997 to 2011, the NINDS (1997-2007; 5R01N736708) and the NIMH (2007- present; 5R01MH080647) funded an autism genetics study (Molecular and Genetic Epidemiology of Autism) that was conducted by the John P. Hussman Institute for Human Genomics at the University of Miami Miller School of Medicine and Vanderbilt University. The Prinicipal Investigator is Margaret A. Pericak-Vance, Ph.D. Jonathan L. Haines, Ph.D., of Vanderbilt University Center for Human Genetics Research, is a subcontract Principal Investigator. Co-Investigators are Michael Cuccaro, Ph.D., John R. Gilbert, Ph.D., and Eden R. Martin, Ph.D.

Study 50 – Utah Autism Genetics Project. From 2005 to 2011, the NIMH (R01 MH069359) to study genetics of autism and related phenotypes using nuclear families and extended pedigrees. Principal Investigator was Hilary Coon. We ascertained a total of 360 trios and small nuclear multiplex families and over 100 multi-generation, multiplex families. These extended pedigrees were identified or confirmed using the Utah Population Database (UPDB), a computerized genealogy database (www.hci.utah.edu/groups/ppr/). Information regarding close relative relationships is contained in the repository. More extended pedigree relationship data may be obtained from the PI. For linkage, genotyp-

ing services were provided by the Center for Inherited Disease Research (CIDR) using the 6K Illumina SNP Linkage Panel 12.

Study 65 – The AGP Simplex Collection (TASC) was funded by an award from Autism Speaks and by funding support to the repository development by the NIMH. The principal investigator and co-investigators on this study were Louise Gallagher, Trinity College Dublin; Astrid Vicente, Instituto Gulbenkian de Ciencia, Oeiras; Joseph Buxbaum, Mount Sinai School of Medicine; Peter Szatmari, McMaster University; William McMahon, University of Utah; Michael Cuccaro, University of Miami; James Sutcliffe, Vanderbilt University; Christine Freitag, Klinikum der Johann-Wolfgang Goethe-Universitt, Frankfurt/Main; Sabine Klauck, Deutsches Krebsforschungszentrum (DKFZ), Heidelberg; Veronica Vieland (DCC Director), Research Institute at Nationwide Childrens Hospital, Ohio; Dan Geschwind, AGRE/UCLA, John Nurnberger, University of Indiana; Ed Cook, University of Illinois at Chicago; Raphael Bernier, University of Washington/CPEA.

Most importantly, we thank the families who have participated in and contributed to these studies.

In addition to the NIMH repository, we are grateful to the Autism Consortiums cohort of families that participated in research and for the use of their data and samples. The Autism Consortium funded a study (Phenotypic and Genetic Factors in Autism Spectrum Disorders) from 2007 to 2011. Patients were enrolled though a collaborative network of five hospitals [Boston Childrens Hospital (BCH), the Lurie Family Autism Center at Massachusetts General Hospital (MGH), The Floating Hospital for Children at Tufts Medical Center, Boston Medical Center, and UMass Medical Center] and phenotyped at either BCH or MGH.
